# Dysregulated Kras/YY1/ZNF322A/Shh transcriptional axis enhances neo-angiogenesis to promote lung cancer progression

**DOI:** 10.7150/thno.47491

**Published:** 2020-08-08

**Authors:** Che-Chung Lin, I-Ying Kuo, Li-Ting Wu, Wen-Hui Kuan, Sheng-You Liao, Jayu Jen, You-En Yang, Cheng-Wei Tang, Yi-Rong Chen, Yi-Ching Wang

**Affiliations:** 1 Department of Pharmacology, College of Medicine, National Cheng Kung University, Tainan, 70101, Taiwan.; 2 Institute of Basic Medical Sciences, College of Medicine, National Cheng Kung University, Tainan, 70101, Taiwan.; 3 Institute of Molecular and Genomic Medicine, National Health Research Institutes, Miaoli, 35053, Taiwan.

**Keywords:** Lung cancer, Kras, Shh, transcription, angiogenesis

## Abstract

Angiogenesis enhances cancer metastasis and progression, however, the roles of transcription regulation in angiogenesis are not fully defined. ZNF322A is an oncogenic zinc-finger transcription factor. Here, we demonstrate a new mechanism of *Kras* mutation-driven *ZNF322A* transcriptional activation and elucidate the interplay between ZNF322A and its upstream transcriptional regulators and downstream transcriptional targets in promoting neo-angiogenesis.

**Methods:** Luciferase activity, RT-qPCR and ChIP-qPCR assays were used to examine transcription regulation in cell models. *In vitro* and *in vivo* angiogenesis assays were conducted. Immunohistochemistry, Kaplan-Meier method and multivariate Cox regression assays were performed to examine the clinical correlation in tumor specimens from lung cancer patients.

**Results:** We validated that Yin Yang 1 (YY1) upregulated *ZNF322A* expression through targeting its promoter in the context of *Kras* mutation. Reconstitution experiments by knocking down YY1 under Kras^G13V^ activation decreased Kras^G13V^-promoted cancer cell migration, proliferation and ZNF322A promoter activity. Knockdown of YY1 or ZNF322A attenuated angiogenesis *in vitro* and *in vivo.* Notably, we validated that ZNF322A upregulated the expression of *sonic hedgehog (Shh)* gene which encodes a secreted factor that activates pro-angiogenic responses in endothelial cells. Clinically, ZNF322A protein expression positively correlated with Shh and CD31, an endothelial cell marker, in 133 lung cancer patient samples determined using immunohistochemistry analysis. Notably, patients with concordantly high expression of ZNF322A, Shh and CD31 correlated with poor prognosis.

**Conclusions:** These findings highlight the mechanism by which dysregulation of Kras/YY1/ZNF322/Shh transcriptional axis enhances neo-angiogenesis and cancer progression in lung cancer. Therapeutic strategies that target Kras/YY1/ZNF322A/Shh signaling axis may provide new insight on targeted therapy for lung cancer patients.

## Introduction

Kras, encode by *Kirsten rat sarcoma viral oncogene* (chromosome 12p12.1), is one of the RAS small GTPase family proteins, which also include Hras and Nras 1,2. Kras protein switches between GTP-bound (active) and GDP-bound (inactive) forms. *Kras* mutations, which predominately occur at codon 12, 13, or 61, can lead to Kras proteins with impaired GTPase activity, resulting in constitutive activation of downstream signaling pathways, and therefore contributes to tumor formation 3,4. Importantly, integrative studies using clinical databases and genetically engineered mouse models showed that *Kras* mutation upregulated expression of FOSL1 to commit a transcriptional program including genes involved in mitosis progression to promote lung and pancreatic cancer progression 5,6. In addition, Kras activation enhances NFκB (p65) expression and its transcription activity in endometrial cancer 7. Importantly, Kras and NFκB concomitantly induce expression of Yin Yang 1 (YY1) transcription factor in pancreatic cancer 8. Moreover, transcription of multiple effectors in the Kras pathway can be modulated by microRNAs 9,10. It is important to unveil more transcription factors downstream of Kras pathway in lung cancer, a disease strongly associated with Kras dysfunctions. Of note, our transgenic mice model showed that mice harboring *kras^G12D^*/*znf322a* double transgenes possessed higher tumor initiating ability compared to those with *kras^G12D^* single transgene.

ZNF322A, also known as ZNF388 or ZNF489, is a zinc-finger transcription factor consisting of 11 Cys_2_His_2_ type krüppel-like zinc-finger motifs 11. Our previous study showed that ZNF322A overexpression promotes lung tumor growth, metastasis and stemness properties partially through activating promoter activity of *alpha-adducin* and *cyclin D1*, while suppressing promoter activity of *p53* and *c-Myc* 12,13. In addition, we found that deregulation of CK1δ-GSK3β-FBXW7α protein degradation system or activation of EGFR-AKT signaling axis results in prolonged ZNF322A stability and transcription activity promoting lung cancer progression 14,15. In our attempt to identify important transcriptional target genes of ZNF322A by integrating our chromatin-immunoprecipitation sequencing (ChIP-seq) and RNA sequencing (RNA-seq) datasets 13, we observed that ZNF322A downstream targets are significantly enriched in vasculature development and angiogenesis. However, the molecular basis for the interaction between ZNF322A and neo-angiogenesis in the context of Kras activation remain poorly defined.

Some well-known signaling axes and genes have been reported to participate in angiogenesis, such as interleukin-8 (IL-8)/CXC chemokine receptors1/2 pathway, NOTCH/delta-like-4 signaling axis and vascular endothelial growth factor (VEGF)/hypoxia induced factor 1 alpha (HIF1α) signaling axis 16-18. Here we show that YY1 transcription factor is a crucial mediator between Kras and ZNF322A in enhancing lung cancer progression. Moreover, our data from lung cancer cell, animal and clinical models demonstrate that sonic hedgehog (Shh) is a downstream transcriptional target of ZNF322A for promoting angiogenesis, and imply Kras/YY1/ZNF322A/Shh transcriptional axis as a previously unknown mechanism contributing to neo-angiogenesis.

## Materials and Methods

### Generation of lung specific transgenic mice

All mouse studies were approved by the Institutional Animal Care and Use Committees of National Health Research Institutes (Permit Number: #101045A) and National Cheng Kung University (Permit Number: #106068) and were performed in accordance with relevant guidelines. To generate lung-specific *znf322a* transgenic mice, pcDNA4/TO/myc-His B expression vector (Invitrogen) was used as a backbone for the construction of the transgenic fragment. The order of the DNA fragments and corresponding franking enzyme sites on the transgenic construct are: Surfactant Protein A (*SPA*) promoter (*Mlu I*/*Hind III*), intron (*Hind III*/*Kpn I*), human *ZNF322A* (*Kpn I*/*Xho I*), and polyA sequence (*Xba I*/*Sac II*). The whole transgenic fragment was excised by *Mlu I* and *Pme I* digestion followed by purification and pronuclear injection of fertilized C57B/J6 mice oocytes. *Znf322a*-transgenic mice were identified by PCR analysis of genomic DNA isolated from tail biopsies. The presence of transgene was determined using the following primers: SPA-Forward primer (5'- TACAGCTCCTGGGCAACGTG -3') and SPA-Reverse (5'- TTGCTTGCATTCAAGGCACTG -3'), yielding a 292 bp PCR product.

Lung specific Tet-on *Kras^G12D^* C57B/J6 transgenic mice (Scgb1a1-rtTA/TetO-Kras4b^G12D^) was a gift from Dr. Ming-Derg Lai (Department of Biochemistry and Molecular Biology, National Cheng Kung University, Taiwan). Reverse tetracycline trans-activator (rtTA) protein was expressed under the control of Scgb1a1 (secretoglobin, family 1A, member 1) promoter. Doxycycline induces rtTA binding to tetracycline operator element, and subsequently promoted *Kras^G12D^* expression. *Kras^G12D^* mice were then crossed with *znf322a* lung-specific mice to create *Kras^G12D^/znf322a* double transgenic mice. All mice with a positive genotype and the control mice were maintained in the animal facility with continual observation till the appearance of disease phenotypes or at the indicated time points. Paraffin blocks of tumors were collected for hematoxylin and eosin (H&E) stain.

### Cell lines and culture conditions

Human lung cancer cell lines H1299 and H460 cells were purchased from ATCC. Human umbilical vein endothelial cells (HUVECs) were kindly provided by Dr. Li-Wha Wu (Institute of Molecular Medicine, National Cheng Kung University, Taiwan). HUVECs seeded in dishes, which were coated with 0.1% gelatin for 1 h, were routinely maintained in endothelial cell growth medium-2 (EGM-2) with addition of growth factors (Lonza). All cell lines were authenticated by the Bioresource Collection and Research Center (Hsinchu, Taiwan) using short tandem repeat profiling (AmpFLSTR Identifiler Plus PCR Amplification Kit). Only mycoplasma negative cells were used.

### Plasmids, RNAi and transfection

Plasmids used in this study are listed in Table S1. siGENOME SMARTpool siRNAs against *ZNF322A* were purchased from Dharmacon; siRNAs against *Shh* was purchased from Thermo Fisher; shRNA clones against *YY1* (KH00440H, Qiagen) were obtained from Dr. Hsin-Ling Hsu (Institute of Molecular and Genomic Medicine, National Health Research Institutes, Taiwan). HA-tagged Kras^G13V^ and Kras^S17N^ plasmids were kindly provided by Dr. Hsiao-Sheng Liu (Department of Microbiology and Immunology, National Cheng Kung University, Tainan). Plasmid and siRNA transfections were carried out using TurboFect (Thermo Fisher) and Lipofectamine 2000 (Invitrogen) reagent according to manufacturer's protocol.

### Promoter constructs and site-directed mutagenesis

*ZNF322A* promoter region (-529 to +223 of the transcriptional start site, TSS) was inserted into the *Kpn*I and *Hind*III sites of pGL4.17 luciferase expression vector. Deletion of two YY1-binding sites within *ZNF322A* promoter (-129 to +223 of the TSS) and mutations of 3-mer of ZNFS22A-motif within *Shh* promoter regions (from TGAGGTCAGGA**GTT**CGAGACCAGCCTGCC to TGAGGTCAGGA**ACC**CGAGACCAGCCTGCC; mutations are shown as underlined letters) were generated by site-directed mutagenesis using indicated wild-type promoter vectors and specific primers listed in Table S2.

### Chromatin immunoprecipitation-quantitative polymerase chain reaction (ChIP-qPCR) and quantitative reverse transcriptase-polymerase chain reaction (RT-qPCR) assay

ChIP was performed in H1299, H1299 Kras^G13V^ and H460 Kras^Q61H^ lung cancer cells manipulated for YY1 or ZNF322A. Lung cancer cells (1.5 × 10^6^ cells) seeded in a 10 cm dish were cross-linked followed by preparation of nuclear lysates using Magna ChIP^TM^ protein G Kit (Millipore). Nuclear lysates were sonicated to shear DNA to around 200~300 bp followed by immunoprecipitation for 16 h at 4 °C using IgG, anti-YY1 or anti-HA-ZNF322A antibody listed in Table S3. Primers for PCR assay of ChIP samples and RT-qPCR reactions are listed in Table S2.

### Transwell migration assay of lung cancer cells or HUVECs

For transwell migration assay of lung cancer cells, 5 × 10^5^ cells were placed in the upper chamber of transwell (Falcon). DMEM medium containing 20% FBS was added to the lower chamber as chemoattractants and the cells were incubated at 37 °C for 12 h. For HUVECs migration, HUVECs (1 × 10^5^) were placed in the upper chamber with serum-free EGM2 while the lower chamber was filled with conditioned medium derived from 1 × 10^5^ lung cancer cells with EGM-2 medium at 1:1 ratio as chemoattractants and incubated at 37 °C for 24 h. The cells attached on the reverse side of the membrane were stained with crystal violet and counted under inverted microscope (Nikon E400, Tokyo, Japan) with randomly selected 10 fields.

### Conditioned medium (CM) preparation, tube formation assay and* in vivo* Matrigel plug angiogenesis assay

Lung cancer cells expressing control, sh*YY1*, si*ZNF322A*, si*Shh*, ZNF322A expression vector, or reconstitution of si*Shh* in ZNF322A (ZNF322A/si*Shh*) were used for CM preparation. Serum-free CM were prepared from culturing lung cancer cells (1 × 10^6^ cells in each 10 cm dish) with 5 mL EBM-2 medium for 30 h. The cell viability was ascertained using the trypan blue dye exclusion assay and was > 98%. The media were collected and centrifuged using Amicon Ultra centrifugal filter units (Millipore) at 800 rpm for 5 min to remove cell debris and then at 3,000 rpm for 5 h at 4 °C to concentrate the CM.

HUVECs were seeded onto 48-well culture dishes coated with 100 μL of Matrigel (13.4 mg/mL; BD Biosciences) at a density of 1.2 × 10^4^ per well. The seeded HUVECs were further treated with CM prepared from lung cancer cells at 37 °C for 6-8 h to allow tube formation. Six random views were photographed and quantified under an upright microscope (Nikon E400). The tube length was quantified using imaging software developed by Dr. Yung-Nien Sun (Department of Computer Science and Information Engineering, National Cheng Kung University, Taiwan).

Matrigel (9 mg/mL; 0.3 mL/mouse) alone or mixed with 50 μL CM derived from different lung cancer cells was injected subcutaneously into the flank of nude mice. On day 10, mice were sacrificed, plugs were removed and fixed in 3.7% formaldehyde/phosphate-buffered saline, paraffin embedded, and slides were immunohistochemically stained for CD31 (endothelial cells marker) and photographed. All mouse studies were approved by the National Cheng Kung University Institutional Animal Care and Use Committee (Permit Numbers: #106068).

### Patient samples and clinical information

A total of 133 surgically resected lung cancer patients were recruited from National Cheng Kung University Hospital after obtaining appropriate institutional review board permission (#A-ER-104-075) and informed consent from the patients. These patients did not receive any anti-angiogenic therapy. The mean follow-up period for these patients was 74 months (range 9-169 months). The histological determinations, including tumor type and disease stage, were performed according to the World Health Organization classification and the TNM classification system, respectively. Information on the sex, age, and smoking history of the patients were obtained from hospital records. Paraffin blocks of tumors were collected for immunohistochemistry.

### Immunohistochemistry assay

Immunohistochemistry was performed to detect protein expression of YY1, ZNF322A, Shh and CD31 in tumor sections from 133 lung cancer patients. Staining of YY1, ZNF322A and Shh was scored as 0 if no cells were stained positive; and scored as 1 if <10% tumor cells were immunostaining-positive; 2 for 10-25%; 3 f or 25-50%; and 4 for >50%. The staining was defined as “high expression” if the staining intensity score was ≥3. The surrounding normal tissue, which shows score 1 served as an internal positive control on each slide. CD31 staining was obtained and quantified using the TissueFax and HistoQuest software (TissueGnostics, Vienna, Austria). The mean staining positive area was calculated within the selected gates: 0.313 mm x 0.175 mm (100 X) for CD31. Six gates were selected in an individual tissue slide. The CD31 staining was graded as “high expression” if staining positive area is greater than 3%. Antibodies used and their experimental conditions are listed in Table S3**.**

### Statistical analysis

Pearson's χ^2^ test was used to compare the correlation of YY1, ZNF322A, Shh and CD31 expression in lung cancer patients. Overall and progression-free survival curves were calculated according to the Kaplan-Meier method using the log-rank test. Cox regression comparison was performed to analyze the relative risk for the patient poor outcome. Quantification of the immunoblotting was analyzed using ImageJ software. Three independent experiments for cell studies and five mice per group for animal studies were analyzed unless indicated otherwise. The scripts used for the analysis are available upon request. Two-tailed Student's t-test was used in cell and animal studies. Data represent mean ± SEM. The levels of statistical significance were expressed as *P*-values, *P < 0.05; ** P < 0.01; *** P < 0.001.

## Results

### *Kras* mutation promotes ZNF322A expression at the transcription level

**Figure 1:**
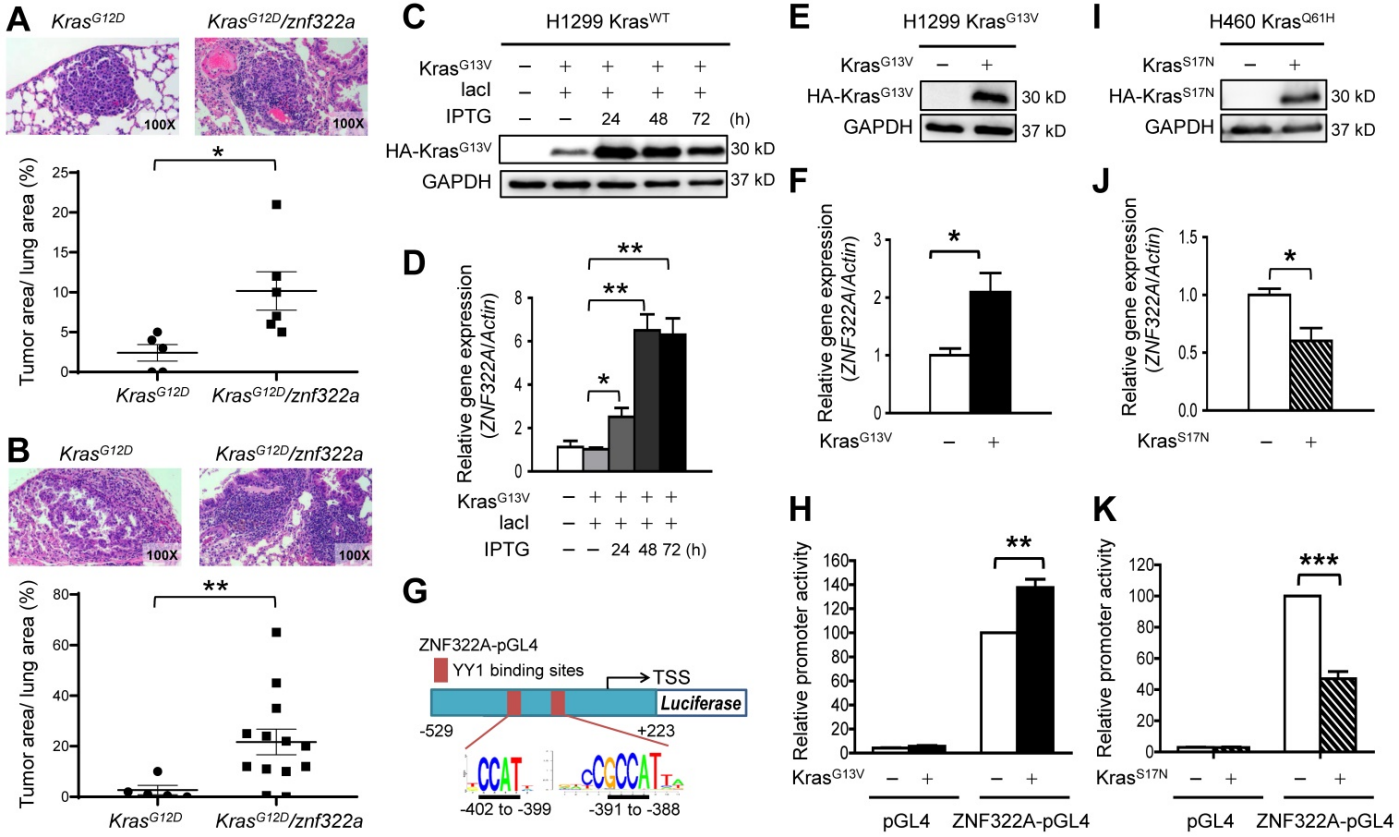
** Oncogenic Kras upregulated *ZNF322A* mRNA expression and promoter activity. A** and** B,** ZNF322A synergized *Kras* mutation-driven lung tumorigenesis *in vivo* in four months (A) or six months (B). The H&E stain results (*Upper*) and the scatter plot diagram (*Lower*) are shown.** C** and** D,** Constitutively-active Kras^G13V^ promoted *ZNF322A* transcription in a dose-dependent manner. Immunoblots confirmed IPTG-induced ectopic overexpression of HA-Kras^G13V^ in H1299 Kras^WT^ cells (C). qRT-PCR revealed that *ZNF322A* mRNA expression was upregulated by Kras^G13V^ overexpression (D). **E** and** F,** Stable Kras^G13V^ expression promoted *ZNF322A* transcription in H1299 Kras^G13V^. Immunoblotting of Kras^G13V^ (E) and *ZNF322A* mRNA expression (F) are shown.** G,** Promoter region (-529~+223) of *ZNF322A*. YY1 binding elements were identified by motif analysis using PWM software. The predicted sequences of the two YY1 binding sites in the *ZNF322A* promoter are as indicated below the map.** H,** Promoter activity assay was performed using ZNF322A-pGL4 promoter in H1299 Kras^WT^ cells.** I**-**K,** Dominant-negative Kras^S17N^ mutation attenuated *ZNF322A* transcription. Immunoblotting of Kras^S17N^ (I), *ZNF322A* mRNA expression (J) and *ZNF322A* promoter activity (K) are shown. Data are presented as mean ± SEM and normalized to the control group (-).* P*-values determined using two-tailed Student's *t*-test. *P< 0.05; ** P< 0.01; *** P< 0.001.

Our previous study identified ZNF322A as an oncogenic transcription factor, which promotes cancer progression by transcriptionally dysregulating downstream cancer-related genes. To further investigate the role of ZNF322A in lung tumorigenesis, we generated *znf322a* lung-specific transgenic mice using C57BL/6 mice. However, we did not observe obvious tumor initiation in the lung area of *znf322a* transgenic mice (Figure S1). Since* Kras* mutations predominantly occur at codon 12 and occasionally at codons 13 and 61 19, we then crossed *znf322a* transgenic mice with *Kras^G12D^* lung-specific transgenic mice to generate *Kras^G12D^/znf322a* double transgenic mice. Notably, mice harboring *Kras^G12D^/znf322a* double transgenes possessed higher tumor initiating ability compared to those with *Kras^G12D^* single transgene after doxycycline-induced *Kras^G12D^* expression, i.e., precancerous adenomas at four months (**Figure 1A**) and advanced adenocarcinoma at six months (**Figure 1B**).

To confirm the positive correlation between *Kras* mutations and ZNF322A expression was not limited to *Kras^G12D^* mutation, we examined Kras-mediated ZNF322A expression in cell lines harboring *Kras* mutation at codon 13 or 61. To this end, we adapted cell culture systems with IPTG-induced constitutively active Kras^G13V^ in H1299 cell line harboring wild-type* Kras* gene (H1299 Kras^WT^). Western blotting results confirmed that IPTG successfully induced ectopic overexpression of Kras^G13V^ (**Figure 1C**). RT-qPCR revealed that *ZNF322A* mRNA expression was upregulated by Kras^G13V^ activation (**Figure 1D**). We then established H1299 cell line stably expressing Kras^G13V^ (H1299 Kras^G13V^) (**Figure 1E**) and found that *ZNF322A* mRNA expression was increased upon Kras^G13V^ overexpression (**Figure 1F**). To further investigate whether Kras activation could drive *ZNF322A* transcription, we identified binding sites of YY1, which is the candidate mediator between Kras and ZNF322A (as described in next section), within the *ZNF322A* promoter. Two putative YY1 binding sites (5'-CCGCCATNTT-3') within the first 500 bp (-402 to -399; -391 to -388) of the *ZNF322A* promoter were identified using the PWM tool (available at https://ccg.epfl.ch/pwmtools/pwmscan.php). We thereby inserted *ZNF322A* promoter region [-529 to +223 of the TSS] into pGL4.17 vector to generate ZNF322A-pGL4 (**Figure 1G**). The luciferase reporter assay results confirmed that Kras^G13V^ activation enhanced *ZNF322A* promoter activity (**Figure 1H**)**.**

To further verify that Kras activated *ZNF322A* transcription, we overexpressed dominant negative Kras^S17N^ in H1299 Kras^WT^ and H460 Kras^Q61H^ (endogenous *Kras^Q61H^* activated mutation) cell lines. Western blotting confirmed the overexpression of dominant negative Kras^S17N^ (**Figure 1I**; Figure S2A). Notably, *ZNF322A* mRNA expression and promoter activity were reduced upon overexpression of dominant negative Kras^S17N^ in H460 Kras^Q61H^ and H1299 Kras^WT^ cell lines (**Figure 1J** and** 1K**; Figure S2B-S2C). Collectively, these results of constitutively active and dominant negative Kras experiments suggested that active *Kras* mutation positively regulates* ZNF322A* transcription.

### YY1 regulates ZNF322A transcription under *Kras* mutation

**Figure 2:**
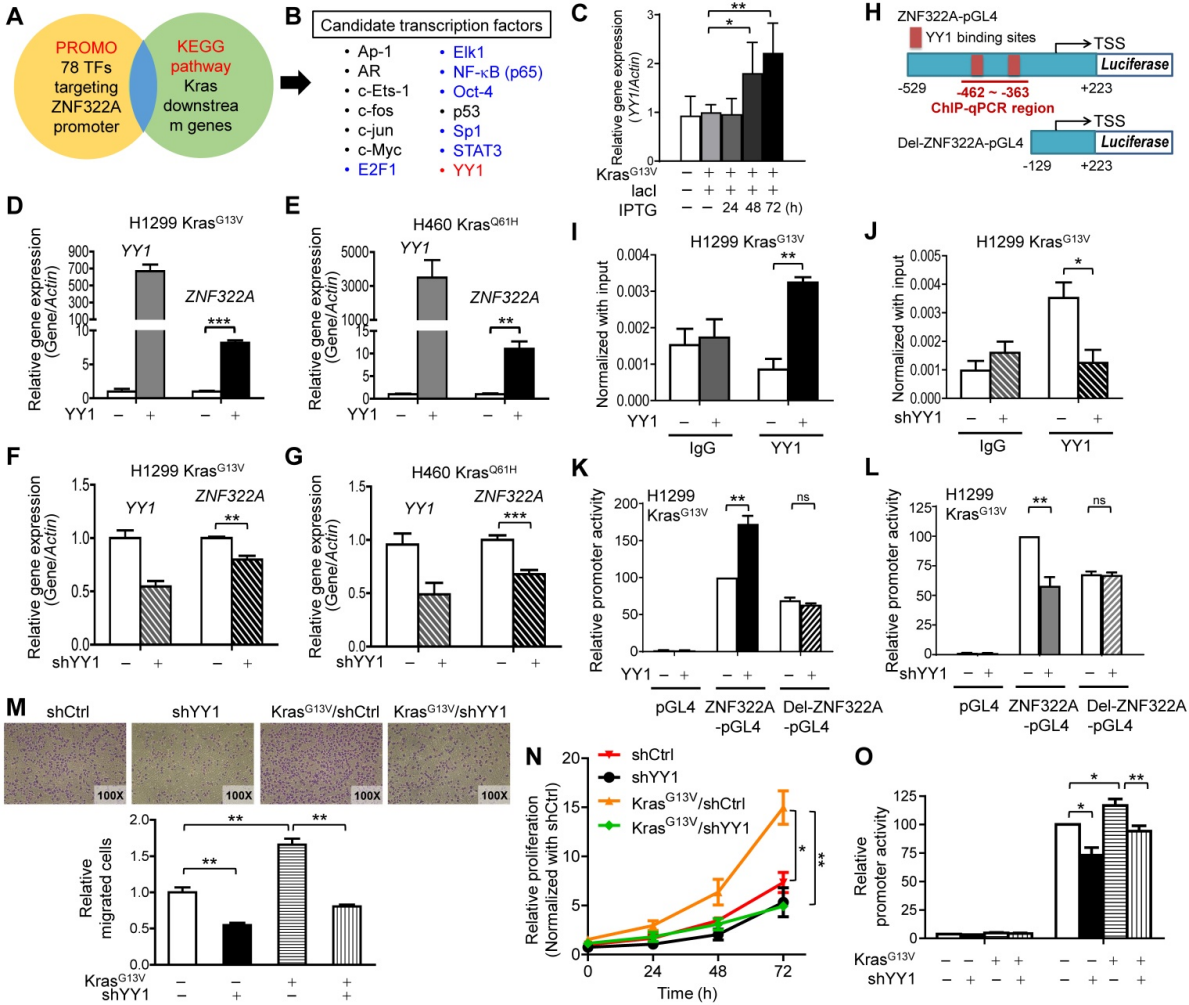
** YY1 positively regulated *ZNF322A* transcription to promote cell migration and proliferation. A** and** B,** Transcription factor (TF) prediction database PROMO revealed 78 TFs may bind to *ZNF322A* promoter. These TFs were further mapped with Kras pathway using KEGG Mapper (KEGG website) (A). The 14 overlapping TFs were considered as candidate transcriptional mediators between Kras and ZNF322A and seven of them were validated for *ZNF322A* transcription (*colored*, B). **C,** RT-qPCR revealed that *YY1* mRNA expression was upregulated by Kras^G13V^ in a dose-dependent manner. **D-G,** RT-qPCR analysis revealed that YY1 promoted *ZNF322A* mRNA expression in H1299 Kras^G13V^ (D) and H460 Kras^Q61H^ (E), while shYY1 reduced *ZNF322A* mRNA expression (F and G). **H,** ChIP-qPCR primers were designed in -462~-363 region of *ZNF322A* promoter as indicated below the map. Sequences of the wild-type and deletion (Del) promoters with deletion of two YY1 binding sites are shown. **I** and** J,** YY1 bound to *ZNF322A* promoter. ChIP assay was performed using anti-YY1 antibody in H1299 Kras^G13V^ overexpressing YY1 (I) or knockdown of YY1 (J). IgG was used as negative control. **K** and** L,** YY1-mediated *ZNF322A* promoter activation was abolished using Del-ZNF322A-pGL4 promoter upon overexpression of YY1 (K) or knockdown of YY1 (L). **M-N,** K-ras/YY1 enhanced lung cancer cell proliferation and migration *via* promoting *ZNF322A* expression. Kras^G13V^ promoted cancer cell migration (M), cell proliferation (N) and *ZNF322A* promoter activity (O) which were attenuated by shYY1 in H1299 cells. Data are presented as mean ± SEM and normalized to the control group (-).* P*-values determined using two-tailed Student's *t*-test. *P< 0.05; ** P< 0.01; *** P< 0.001.

Next, we searched for the candidate mediators between Kras activation and *ZNF322A* transcription using the PROMO transcription factor (TF) prediction database (available at http://alggen.lsi.upc.es/cgi-bin/promo_v3/promo/promoinit.cgi?dirDB=TF_8.3), and 78 TFs that may bind to *ZNF322A* promoter were revealed. Those TFs were further mapped with Kras pathway (KEGG Mapper, available at http://www.genome.jp/kegg/). The 14 overlapping TFs were considered as the candidate transcription mediators downstream of Kras (**Figure 2A**). To investigate whether these candidate TFs could regulate ZNF322A transcription, we transfected H1299 or H460 cells with expression vectors of seven TFs available in our group. RT-qPCR analysis results revealed that overexpression of E2F1, ELK1, NFκB (p65), Oct4, Sp1 or STAT3 did not affect *ZNF322A* mRNA expression (**Figure 2B**; Figure S3A). Notably, RT-qPCR revealed that *YY1* mRNA expression was dose-dependently upregulated by IPTG-induced constitutively active Kras^G13V^ (**Figure 2C**; Figure S3B). Thus, we focused on YY1 transcription factor.

In order to determine whether YY1 regulated *ZNF322A* transcription, we ectopically overexpressed YY1 in H1299 Kras^G13V^ and H460 Kras^Q61H^ cell lines. Results of RT-qPCR analysis demonstrated that *ZNF322A* mRNA expression was significantly upregulated by YY1 (**Figure 2D** and **2E**), while knockdown of YY1 (sh*YY1*) reduced *ZNF322A* mRNA expression (**Figure 2E** and **2F**) in H1299 Kras^G13V^ and H460 Kras^Q61H^ cells. The immunoblotting results confirmed the expression of YY1 protein upon overexpression or knockdown of YY1 in H1299 Kras^WT^, H1299 Kras^G13V^ and H460 Kras^Q61H^ cell lines (Figure S4A and S4B).

Next, we performed ChIP-qPCR at *ZNF322A* promoter region (-462~-363) which contained two YY1-binding sites (**Figure 2H**) to confirm that YY1 indeed binds to *ZNF322A* promoter region (**Figure 2I**). Knockdown of YY1 significantly attenuated its ability to bind the* ZNF322A* promoter, validating that the ChIP-qPCR results observed in Figure 2I was a true YY1 binding signal (**Figure 2J**). To further verify whether YY1 regulated the activity of *ZNF322A* promoter, luciferase promoter activity assay using ZNF322A-pGL4 (-529 to +223 of the TSS) and Del-ZNF322A-pGL4 (-129 ~ +223 with deletion of two YY1 binding sites at -402 ~ -388) (**Figure 2H**) were performed. As shown in **Figure 2K** and **2L**, overexpression of YY1 increased promoter activity of the ZNF322A-pGL4 promoter, while YY1-mediated *ZNF322A* promoter activity was completely abolished when Del-ZNF322A-pGL4 promoter deleted for the two YY1 sites was used. In agreement, knockdown of YY1 reduced promoter activity of ZNF322A-pGL4, but not for Del-ZNF322A-pGL4 promoter. These results suggested that -462 ~ -363 regions in *ZNF322A* promoter contained the binding sites for YY1.

### Kras/YY1 enhances lung cancer cell proliferation and migration *via* promoting ZNF322A transcription* in vitro*

Since we unveiled YY1 as a crucial mediator of Kras mutation-driven transcription of ZNF322A, we analyzed the role of YY1 in lung cancer cell proliferation and migration. Transwell migration assay showed that knockdown of YY1 significantly reduced cell migration promoted by Kras^G13V^ (**Figure 2M**). Consistently, Kras^G13V^ activation promoted cell proliferation, which was abolished by YY1 ablation (**Figure 2N**). Promoter activity assays validated that Kras^G13V^-activated *ZNF322A* promoter activity was attenuated by *YY1* knockdown (**Figure 2O**). Collectively, these results supported that ZNF322A upregulation mediated by Kras/YY1 axis promotes proliferation and migration of lung cancer cells.

### ZNF322A regulates mRNA expression of genes involved in angiogenesis

**Figure 3:**
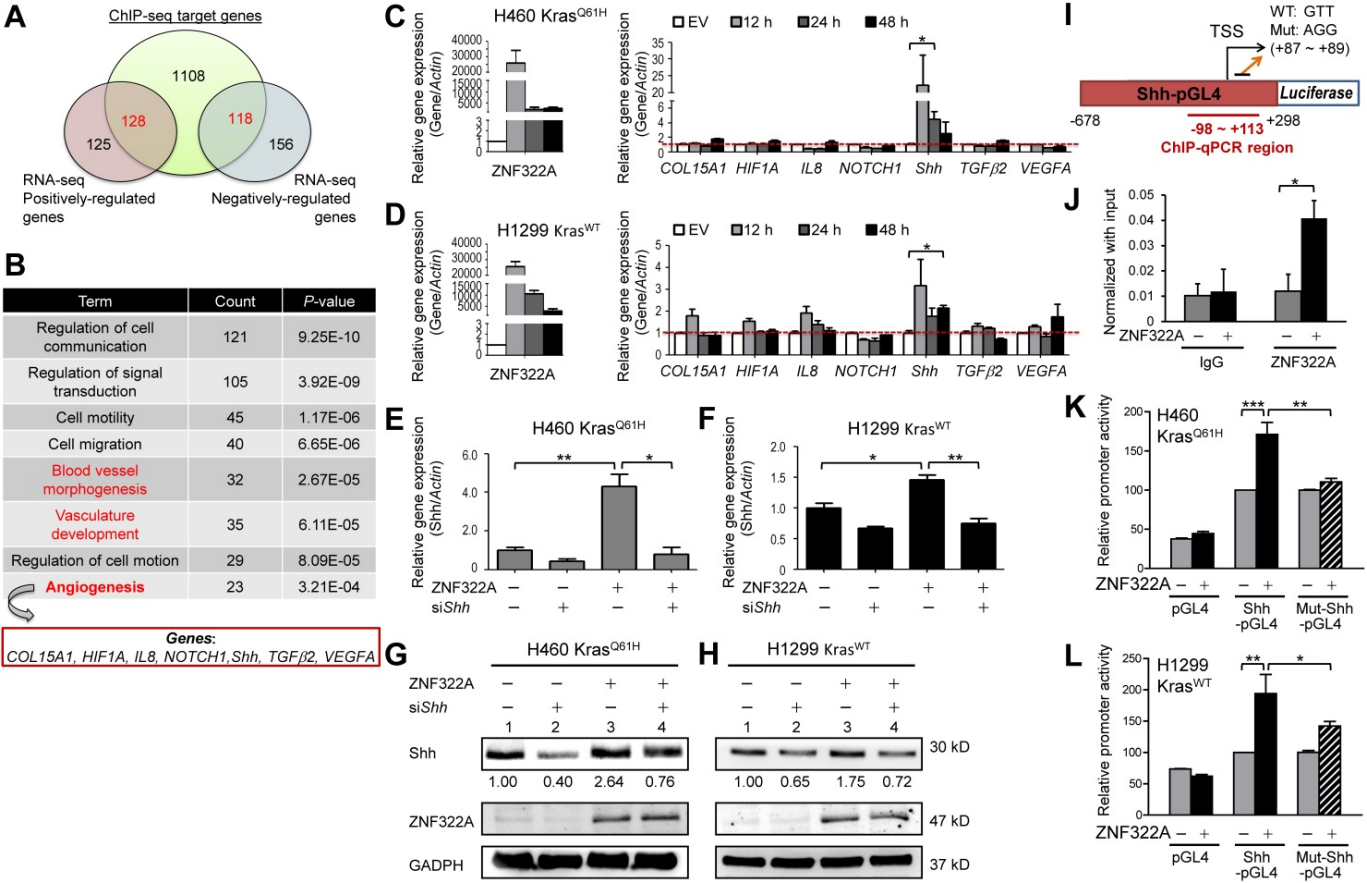
ZNF322A transcriptionally activated *Shh* promoter. **A** and **B,** Integrated ChIP-seq and RNA-seq analysis revealed the novel role of ZNF322A in angiogenesis. ZNF322A-mediated transcriptome (A) and pathway analysis using DAVID software are shown.** C** and **D,** RT-qPCR validation of the seven angiogenic genes identified using DAVID. Only the *Shh* mRNA level was increased in ZNF322A-overexpressing H460 Kras^Q61H^ (C) and H1299 Kras^WT^ (D) lung cancer cells at 12, 24 and 48 h time points. **E**-**H,** Reconstitution experiments showed that Shh acted as a downstream factor of ZNF322A in lung cancer cells. RT-qPCR (E and F) and immunoblots (G and H) confirmed that Shh expression level was decreased in ZNF322A/si*Shh* group compared with ZNF322A group (groups 4* vs.* 3) in H460 Kras^Q61H^ and H1299 Kras^WT^ lung cancer cells. Normalized Shh protein fold changes are as indicated below the blots. **I,** Promoter region (-678~+298) of *Shh*. ChIP-qPCR primers designed in -98~+113 region of *Shh* promoter are labeled below the map. Sequences of the wild-type (WT) and mutated (Mut) promoters are shown (+87 ~ +89). **J,** ZNF322A bound to *Shh* promoter. ChIP assay was performed using anti-HA antibody in H460 Kras^Q61H^ overexpressing ZNF322A. IgG was used as negative control. **K** and** L,** Overexpression of ZNF322A influenced promoter activity of Shh-pGL4 but not Mut-Shh-pGL4 in ZNF322A-overexpressing H460 Kras^Q61H^ (K) and H1299 Kras^WT^ (L) lung cancer cells. Data are presented as mean ± SEM.* P*-values determined using two-tailed Student's *t*-test. *P< 0.05; ** P< 0.01; *** P< 0.001.

We further identified ZNF322A downstream genes by integrating our previous ChIP-seq and RNA-seq datasets (**Figure 3A**). Using DAVID Functional Annotation Clustering Tool (available at http://david.ncifcrf.gov/home.jsp), we found that many of the overlapped genes mapped to the angiogenesis-related pathways, including vasculature development, blood vessel morphogenesis and angiogenesis pathway (**Figure 3B**). Next, RT-qPCR was conducted to validate the mRNA expression level of seven angiogenic genes viz., *COL15A1* (*collagen, type XV, alpha 1*), *HIF1α*, *IL-8*, *NOTCH1*, *Shh*, *TGFβ2*, and* VEGFA* in H460 Kras^Q61H^ and H1299 Kras^WT^ cells overexpressing ZNF322A. Among them, *Shh* mRNA level positively correlated with ZNF322A at all time points (12, 24, and 48 h) examined in both H460 Kras^Q61H^ and H1299 Kras^WT^ cell lines (**Figure 3C** and 3**D**).

### ZNF322A transcriptionally activates the expression of Shh

In order to test whether Shh is a transcription target of ZNF322A, we determined Shh mRNA and protein expression levels in reconstitution experiments by knocking down Shh (si*Shh*) in ZNF322A-overexpressed (ZNF322A) cancer cells. The si*Shh* attenuated the ZNF322A-induced expression of *Shh* mRNA (bars 4 *vs*. 3, **Figure 3E** and **3F**) and Shh protein (lanes 4 *vs*. 3, **Figure 3G** and **3H**). These data suggested that Shh is a downstream effector of ZNF322A-mediated gene expression.

In our previous ChIP-seq study, we have revealed the ZNF322A binding DNA element using the MEME motif analysis 13. As shown in** Figure 3I**, ZNF322A binding sequences were found at +87 ~ +89 on the *Shh* promoter. We then performed ChIP-qPCR to confirm ZNF322A binding at *Shh* promoter region (-98 ~ +113) in H460 Kras^Q61H^ lung cancer cells (**Figure 3J**). Next, we examined whether ZNF322A binding enhanced *Shh* promoter activity by inserting *Shh* promoter region (-678 to +298 of the TSS) into pGL4.17 vector (Shh-pGL4) and then performed luciferase reporter assay combined with site-directed mutagenesis at +87 ~ +89 region by changing GTT to AGG sequences (Mut-Shh-pGL4, **Figure 3I**). Our results showed that ZNF322A overexpression activated Shh-pGL4 promoter activity but marginally changed Mut-Shh-pGL4 promoter activity in both H460 Kras^Q61H^ and H1299 Kras^WT^ lung cancer cells (**Figure 3K** and **3L**). The results confirmed that ZNF322A enhances *Shh* promoter activity and +87 ~ +89 region in *Shh* promoter contains the binding site for ZNF322A.

### Reconstitution experiments showed that ZNF322A/Shh axis increased endothelial cell migration and tube formation *in vitro*

**Figure 4:**
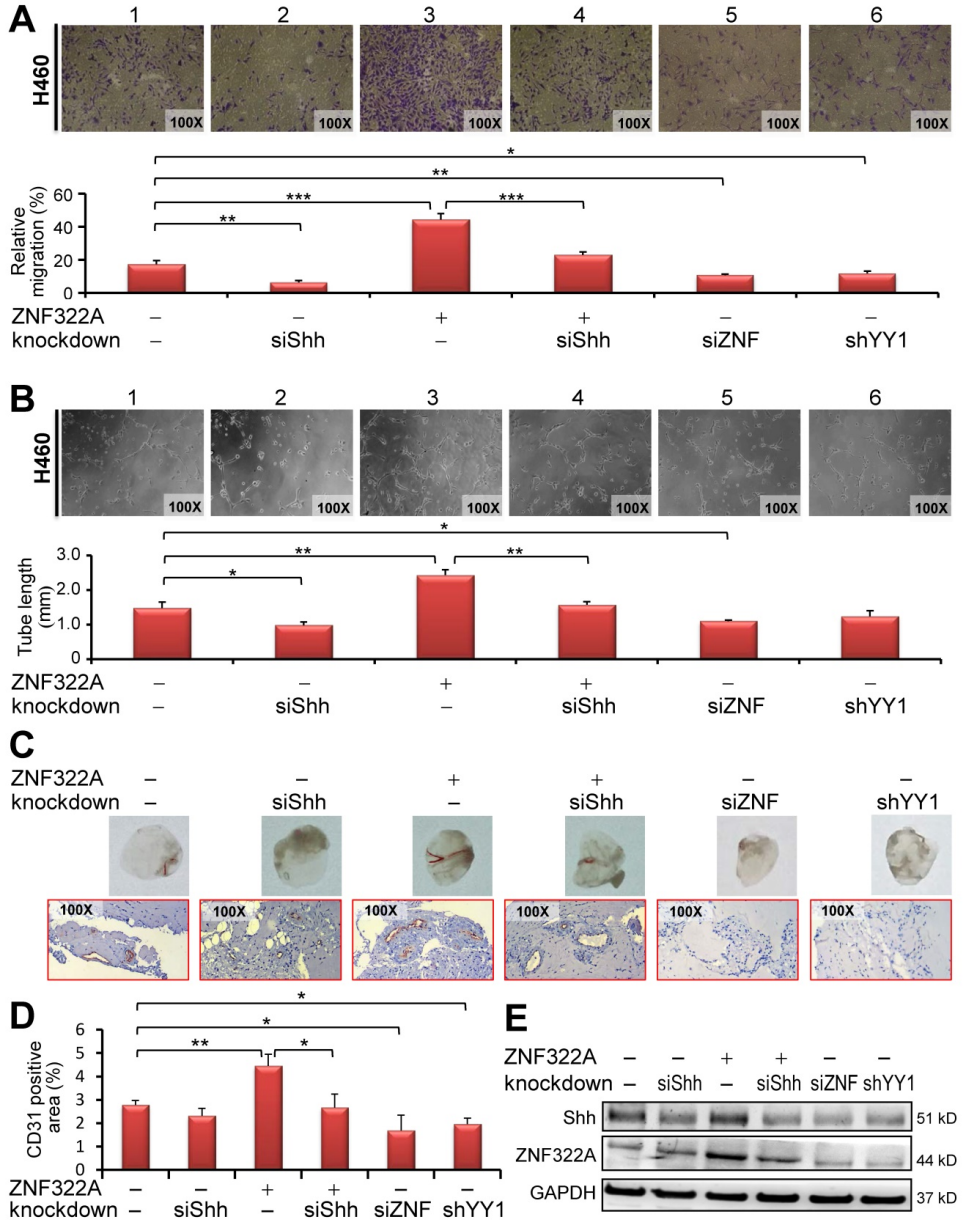
YY1/ZNF322A/Shh axis regulated angiogenesis *in vitro* and *in vivo*.** A** and **B**, Transwell migration assay (A) and tumor formation assay (B) showed that CM derived from si*Shh* in ZNF322A overexpressing (*group 4*) cells inhibited HUVECs migration ability or tumor formation ability compared with CM from ZNF322A-overexpressed (*group 3*) H460 Kras^Q61H^ lung cancer cells. Knockdown of ZNF322A (*group 5*) or YY1 (*group 6*) in H460 Kras^Q61H^ lung cancer cells also attenuated HUVECs migration and tumor formation abilities (panels 5 and 6). siShh was included for comparison (*group 2*). Migration ability was monitored at 24 h with each group quantified by comparison with initial seeding number of HUVECs. The tube formation was monitored at 6-8 h with each group quantified for the tube length. **C-E,** Knockdown of Shh inhibited angiogenesis mediated by ZNF322A overexpression in H460 Kras^Q61H^ lung cancer cells using *in vivo* Matrigel plug angiogenesis assay. (C) Matrigel plug images (*Upper*) and IHC stains (*Lower*) showed that angiogenesis was decreased in Matrigel implants with CM from si*Shh*, ZNF322A/si*Shh,* si*ZNF322A* or shYY1-H460 Kras^Q61H^ cells but not with CM from ZNF322A-overexpressing H460 lung cancer cells compared with those from EV/siCtrl-H460 cells. (D) The angiogenesis of each group was measured by the area of CD31-positive stained cells. (E) Western blot confirmed that Shh and ZNF322A were successfully manipulated in H460 Kras^Q61H^ lung cancer cells before CM collection. *P* values were calculated by two-tailed *t*-test. Data were mean ± SEM. *, *P*<0.05; **, *P*<0.01; ***, *P*<0.001.

Our data so far suggest that ZNF322A transcriptionally activates the pro-angiogenesis gene *Shh* and that YY1 regulates *ZNF322A* gene expression. Therefore, we examined whether activation of YY1/ZNF322A/Shh exerted pro-angiogenesis effects. We performed transwell migration assay and tube formation of HUVECs cultured with the conditioned medium (CM) derived from cancer cells manipulated for YY1, ZNF322A and/or Shh expression level. *In vitro* HUVEC transwell migration (**Figure 4A**) and tube formation (**Figure 4B**) assays showed that si-*Shh* in H460 Kras^Q61H^ lung cancer cells inhibited HUVECs migration (panel 2), whereas overexpression of ZNF322A promoted HUVECs migration (panel 3) compared with control group (panel 1, **Figure 4A** and **4B**). Importantly, migration and tube formation abilities of HUVECs were indeed attenuated when treated with CM derived from reconstituted ZNF322A/si*Shh* lung cancer cells (panel 4) compared with those from ZNF322A overexpressing lung cancer cells (panel 3, **Figure 4A** and **4B**), suggesting that Shh is a downstream effector of ZNF322A-mediated pro-angiogenesis. In addition, knockdown of ZNF322A or YY1 attenuated HUVECs migration ability in CM from H460 Kras^Q61H^ lung cancer cells (panels 5 and 6, **Figure 4A** and **4B**). Similar results were observed in CM from H1299 Kras^WT^ lung cancer cells (Figure S5). These results showed that ZNF322A/Shh axis promotes migration and tumor formation abilities of HUVECs.

### ZNF322A/Shh axis enhanced *in vivo* angiogenesis

We further performed *in vivo* Matrigel plug angiogenesis assay. Mixtures of Matrigel with CM from H460 Kras^Q61H^ lung cancer cells manipulated for expression of YY1, ZNF322A and/or Shh were injected subcutaneously into nude mice and then the Matrigel plugs were collected on day 10 for macroscopic analysis and IHC staining of CD31 to reveal blood vessel infiltration. As shown in **Figure 4C** (upper panel), Matrigel plugs from CM of si*Shh* (panel 2), si*ZNF322A* (panel 5) or sh*YY1* (panel 6) H460 cells showed a decrease of blood vessel-like structure while those from CM of ZNF322A (panel 3) showed an increase of infiltrated blood vessel-like structure. Notably, mice group injected with CM from ZNF322A/si*Shh*-H460 cells (panel 4) showed less angiogenesis than those from ZNF322A-H460 cells (panel 3). In addition, we used IHC to determine the presence of CD31, an endothelial cells marker. CD31-positive infiltration signals were increased in ZNF322A group (panel 3) while less endothelial infiltration was observed for the ZNF322A/si*Shh,* si*Shh*, si*ZNF322A* and sh*YY1* groups (lower panel,** Figure 4C**). Quantitative results are shown in **Figure 4D**. Western blot confirmed that ZNF322A and si*Shh* were successfully manipulated in lung cancer cells before CM collection (**Figure 4E**). These *in vivo* results corroborated with the *in vitro* data, indicating that ZNF322A/Shh axis enhances angiogenesis.

Moreover, we examined whether CD31 endothelial cells marker was increased in tumor xenograft derived from H460 Kras^Q61H^ lung cancer cells manipulated for ZNF322A expression level. IHC data revealed that CD31 signal was increased in ZNF322A overexpression group, and decreased in ZNF322A knockdown group compared to control group (Figure S6). Altogether, the results were consistent with the scenario that endothelial migration and angiogenesis abilities were promoted by ZNF322A, in part through promoting the expression of Shh, a pro-angiogenesis factor.

### Positive correlations of ZNF322A, Shh and CD31 expression in lung cancer patients

**Figure 5:**
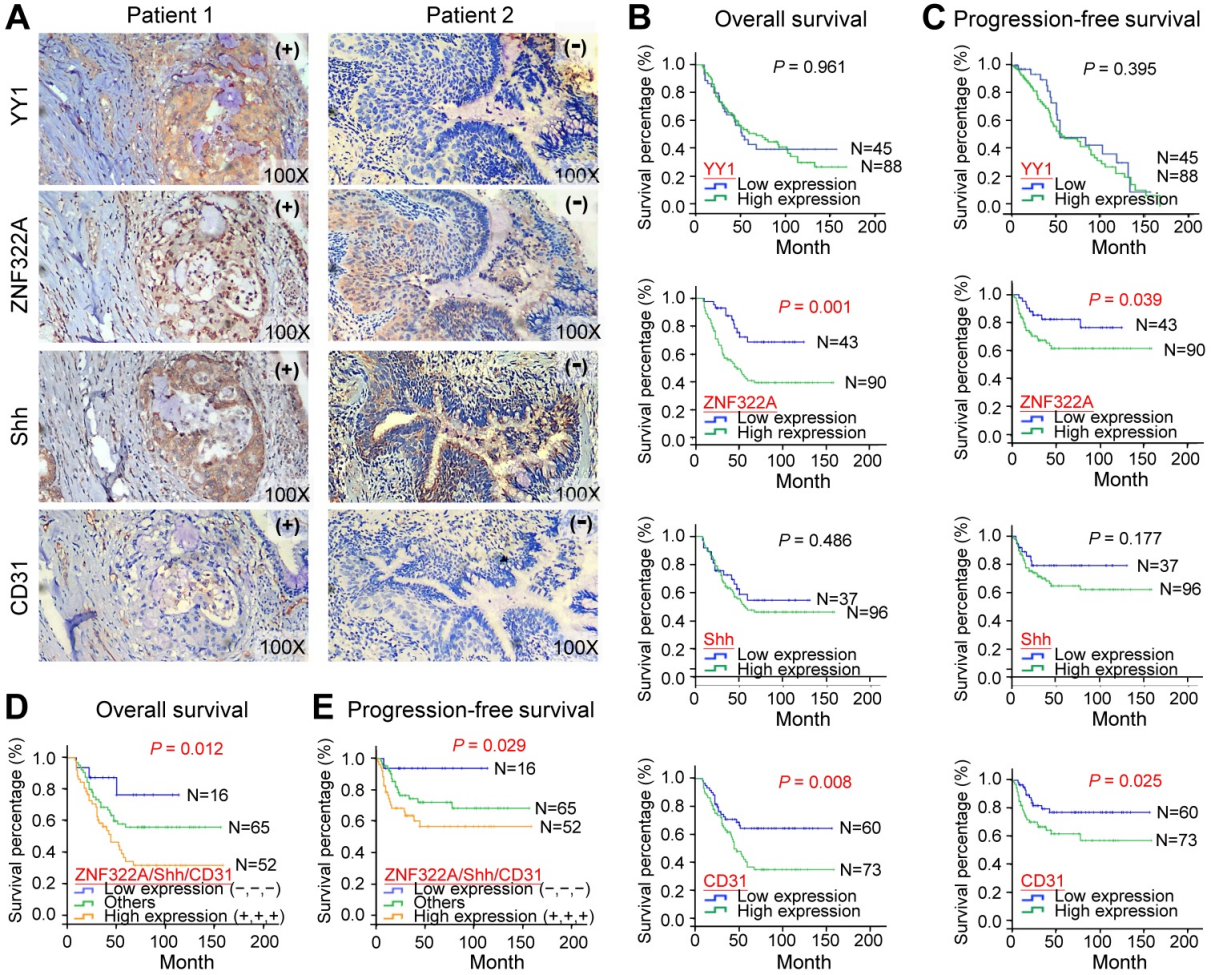
Lung cancer patients with concordant ZNF322A^high^/Shh^high^/CD31^high^ expression profile were associated with poor overall survival and progression-free survival. **A,** IHC images of tumor specimens from two representative lung cancer patients showed that YY1, ZNF322A, Shh and CD31 displayed a concordant expression pattern, -, low cancer baseline expression; +, high expression. **B** and **C,** Kaplan-Meier survival analysis showed that patients with ZNF322A^high^ and CD31^high^ expression had poor overall survival (OS, B) and progression-free survival (PFS, C) in 133 lung cancer patients. **D** and **E,** Lung cancer patients with concordant ZNF322A^high^/Shh^high^/CD31^high^ expression profile were associated with worse OS (D) and PFS (E). *P* values were determined using log-rank test.

Next, we confirmed our proposed YY1/ZNF322/Shh/CD31 axis in clinical samples. We performed IHC analysis to examine the expression of YY1, ZNF322A, Shh and CD31 in surgically resected tumor specimens from 133 lung cancer patients (**Figure 5A**; **Table 1**). The results demonstrated that 67.7% of patients showed high ZNF322A expression which correlated with advanced tumor stage (*P*=0.002; **Table 1**). High Shh expression was found in 72.2% of patients and was also associated with tumor stage (*P*=0.001; **Table 1**). Notably, high ZNF322A expression showed concordantly increased Shh expression and positive CD31 staining (*P*<0.001; **Table 1**). However, high YY1 expression only correlated with squamous cell carcinoma (SCC) patients, while high expression of ZNF322A, Shh, and CD31 (ZNF322A^high^/Shh^high^/CD31^high^) tended to occur more frequently in adenocarcinoma (ADC) patients than in SCC patients (**Table 1**). In addition, we previously reported a positive correlation between high expression of ZNF322A and phosphorylated AKT in more than 80% of lung cancer patients 15. Phosphorylated AKT has been postulated as a secondary event of oncogenic Kras in lung cancer 20,21, indicating Kras activation in this cohort. These clinical correlation data suggested that the ZNF322A/Shh/CD31 axis induced neo-angiogenesis in tumor and associated with advanced lung cancer.

### ZNF322A^high^/Shh^high^/CD31^high^ in lung cancer patients were associated with poor overall survival and progression-free survival

To determine whether the ZNF322A/Shh/CD31 axis was associated with prognosis in human lung cancer, we analyzed overall survival (OS) and progression-free survival (PFS) using the Kaplan-Meier method in 133 patients. Although YY1 and Shh did not show survival prediction potential, overexpression of ZNF322A correlated with poor OS (*P*=0.001; **Figure 5B**) and PFS (*P*=0.039; **Figure 5C**) in lung cancer patients. Moreover, lung cancer patients with concordantly high expression of ZNF322A, Shh, and CD31 (ZNF322A^high^/Shh^high^/CD31^high^) showed the worse OS (*P*=0.012; **Figure 5D**) and PFS (*P*=0.029; **Figure 5E**).

Next, we performed univariate and multivariate Cox regression analyses in this cohort of 133 lung cancer patients. Univariate Cox regression analysis revealed that patients with ZNF322A^high^, CD31^high^ expression profile, late stage, or lymph node metastasis had poor survival outcome (**Table 2**). Importantly, multivariate Cox regression analysis indicated that patients with ZNF322A^high^/CD31^high^ expression profile showed significantly high risk of death (hazard ratio = 3.952, *P* = 0.012; **Table 2**) even after adjusting for the clinical parameters exhibiting potential risk in univariate analysis. These results indicated that the combination of high ZNF322A, high Shh and high CD31 expression could be used as an independent factor in predicting the clinical outcome in lung cancer patients.

## Discussion

**Figure 6:**
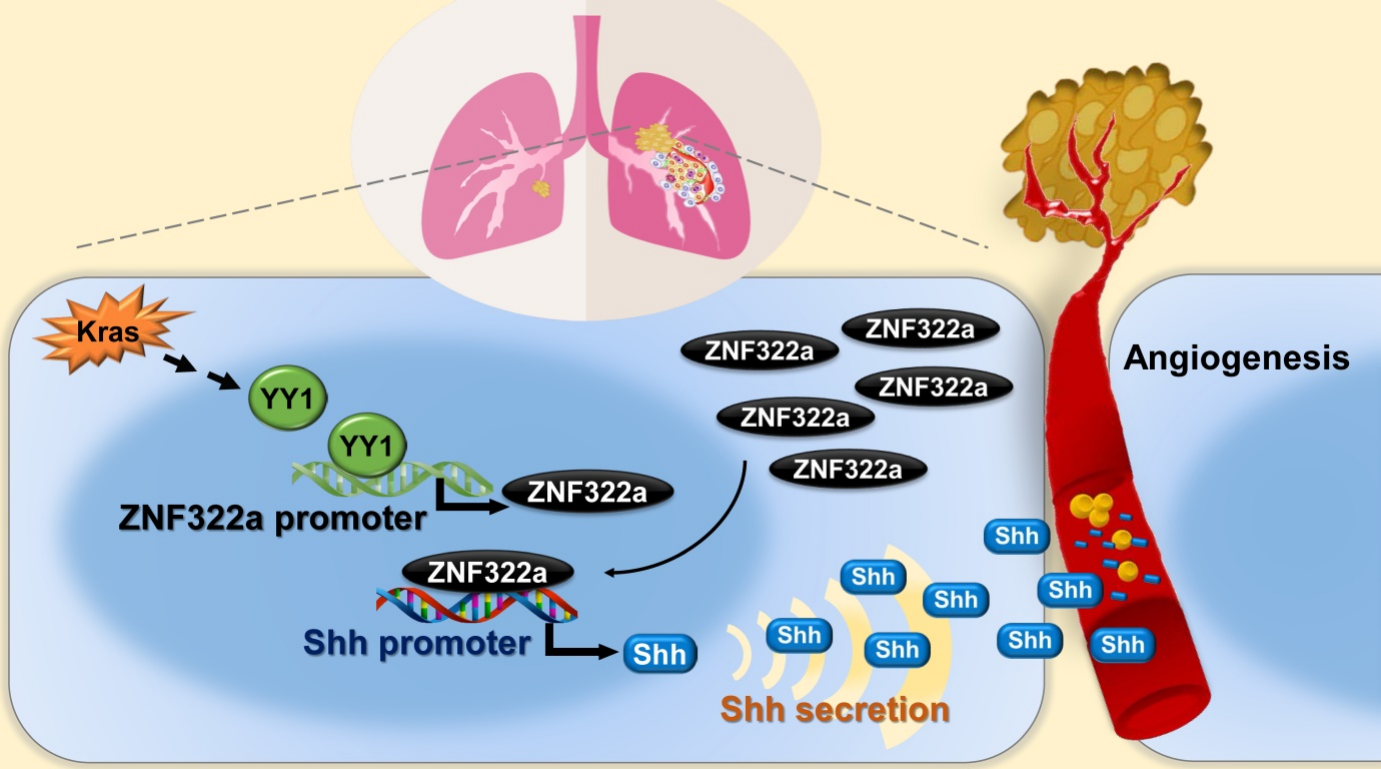
Schematic diagram of Kras/YY1/ZNF322A/Shh transcription axis contributing to neo-angiogenesis in lung cancer model. Oncogenic Kras signaling enhances expression of YY1, the transcription factor that directly activates *ZNF322A* transcription. Subsequently, overexpressed ZNF322A transcription factor binds to *Shh* promoter and enhances its expression. Kras/YY1/ZNF322A-mediated Shh activation promotes angiogenesis abilities *in vitro/vivo*. Clinically, a positive correlation between ZNF322A^high^/Shh^high^/CD31^high^ is found in tumors derived from lung cancer patients with poor prognosis.

In this study, we identify Kras/YY1/ZNF322A/Shh transcriptional axis as part of an important mechanism underlining neo-angiogenesis and lung cancer metastasis. Mechanistically, oncogenic Kras signaling enhances expression of YY1, the transcription factor that directly activates *ZNF322A* transcription. Subsequently, overexpressed ZNF322A transcription factor binds to *Shh* promoter and enhances its expression. Furthermore, we demonstrate that Kras/YY1/ZNF322A-mediated Shh activation promotes angiogenesis abilities *in vitro/vivo*. Clinically, a positive correlation between ZNF322A^high^/Shh^high^/CD31^high^ is found in tumors derived from lung cancer patients with poor prognosis. Our findings not only present a previously undefined regulatory mechanism by which ZNF322A synergizes Kras^G12D^-induced lung tumorigenesis but also indicate that dysregulation of YY1/ZNF322A transcriptional axis promotes expression of angiogenic factor Shh and cancer progression in lung cancer (**Figure 6**).

We discovered that YY1 positively regulated *ZNF322A* expression at the transcriptional level. Overexpression of YY1 enhanced *ZNF322A* mRNA expression and promoter activity of ZNF322A-pGL4, while knockdown of YY1 showed the reverse effects. Such YY1-mediated *ZNF322A* transcription regulation was abolished when the Del-ZNF322A-pGL4 promoter with deletion of two YY1 binding sites at -462 ~ -363 were used for promoter activity assay. YY1 can act either as an oncogene or a tumor suppressor depending on the cell context because of the multiple roles played by YY1 in regulation of transcription 22-24. Previous studies have demonstrated that YY1 interacts with p300, AP-1 or TET-catalyzed chromatin complex to cooperatively regulate downstream gene transcription 25-27. Our RT-qPCR results showed that overexpression of candidate transcription factors E2F1, ELK1, NFκB, Oct4, Sp1 or STAT3 did not influence *ZNF322A* mRNA expression. However, it is still possible that other TF candidates, for example c-jun or c-myc, which has been shown to cooperate with mutated Kras 28-30, may play a role in regulating *ZNF322A* transcription. Whether YY1 need additional TF or transcription co-regulators to drive *ZNF322A* transcription is worthy of further investigation.

The activity of the hedgehog (Hh) pathway is characterized by its dependence on Hh ligands which are produced in secretory cells such as cancer epithelial cell, mural cell, and stromal cell 31-33. These ligands activate downstream signaling in receiving cells such as cancer epithelial cell, fibroblast, and endothelial cell 33, 34, 35. Cancer cells have been shown to express Shh ligands and drive canonical signaling in tumor-associated fibroblasts to promote tumor angiogenesis through paracrine Shh signal to adjacent endothelial cells 36. Although Shh is the most-studied hedgehog so far, only a few studies investigate transcription regulation of *Shh* gene. For example, NF-κB transcriptionally upregulates *Shh* expression in pancreatic carcinoma cells 37. In addition, p63 directly targets and positively regulates the transcription of Shh signaling components such as Shh, Gli2 and Ptch1 to modulate the Shh signaling pathway 38. Recent report shows that nuclear factor (erythroid-derived 2)-like 2 (NRF2) binds to the promoter of Shh to upregulate Shh mRNA and protein levels, which leads to activation of the Shh pathway and resistance to sorafenib in hepatocellular carcinoma 39. Our study revealed a novel mechanism by which ZNF322A enhanced neo-angiogenesis in part by activating Shh expression at the transcription level.

Reconstitution experiments demonstrated that ZNF322A/Shh axis was important for angiogenic activity *in vitro* and *in vivo* in H460 lung cancer cells with endogenous *Kras* mutation. Interestingly, our clinical data indicated significant positive correlations between ZNF322A, Shh and CD31. In addition, ZNF322A, Shh and CD31 were all associated with T-N-M stage. These data suggested that ZNF322A, Shh and CD31 played important roles in lung tumor angiogenesis and metastasis. However, Shh or YY1 overexpression alone did not significantly correlate with poor OS and PFS rates. Since Shh expression pattern examined by IHC showed staining in both cytosolic and extracellular compartments in tumor specimens from lung cancer patients, it is possible that the cytosolic immature Shh proteins were also scored in our IHC result. Similarly, a ubiquitous immunoreactivity of YY1 in tumor samples may account for the absence of correlation with clinical parameters or ZNF322A expression pattern. Nevertheless, the expression profile of ZNF322A^high^/Shh^high^/CD31^high^ is a potential prognostic biomarker for lung cancer and may be for other cancers.

## Conclusion

This study provides new mechanistic insights into how oncogenic Kras-induced YY1/ZNF322A transcriptional axis promotes lung cancer progression. We also demonstrate interplay between ZNF322A/Shh axis by lung cancer epithelial cells and endothelial cells in regulation of neo-angiogenesis. Mechanistically, YY1 transcription activation induced overexpression of oncoprotein ZNF322A, ZNF322A then bound to *Shh* promoter and enhanced its expression. Kras has been considered to be undruggable thus far. Therefore, therapeutic strategies have shifted toward Kras downstream signaling. We proposed that lung cancer patients with the expression profile of ZNF322A^high^/Shh^high^/CD31^high^ may be selected for further treatment with Shh neutralizing antibodies, although targeting Shh *via* antibodies has not reached human trials 40,41. Alternatively, these patients may be treated with drugs targeting Shh signaling effectors such as the SMO antagonists or VEGFR2 inhibitor already approved by the US Food and Drug Administration 42-44. Therapeutic strategies that target Kras/YY1/ZNF322A/Shh signaling axis may provide new insight on targeted therapy for lung cancer patients.

## Supplementary Material

Supplementary figures and tables.

## Figures and Tables

**Table 1:** Alteration of ZNF322A, YY1, Shh and CD31 expression in relation to clinicopathological parameters in 133 lung cancer patient patients^a^

Clinicopathological parameters		ZNF322A protein		YY1 protein		Shh protein		CD31 expression	
	N=133	N=43 (32.3%)	N=90 (67.7%)	N=45 (33.8%)	N=88 (66.2%)	N=37 (27.8%)	N=96 (72.2%)	N=60 (45.1%)	N=73 (54.9%)
	Total	Low (%)	High (%)	Low (%)	High (%)	Low (%)	High (%)	Low (%)	High (%)
**Age**									
<65	71	20 (28.2)	51 (71.8)	26 (36.6)	45 (63.4)	15 (21.1)	56 (78.9), ***P*=0.05**	31 (43.7)	40 (56.3)
≥65	62	23 (37.1)	39 (62.9)	19 (30.6)	43 (69.4)	22 (35.5)	40 (64.5)	29 (46.8)	33 (53.2)
**Sex**									
Male	76	23 (30.3)	53 (69.7)	22 (28.9)	54 (71.1)	25 (21.1)	51 (78.9)	33 (43.4)	43 (56.6)
Female	57	20 (35.1)	37 (64.9)	23 (40.4)	34 (59.6)	12 (32.9)	45 (67.1)	27 (47.4)	30 (52.6)
**Stage**									
I-II	81	34 (42.0)	47 (58.0), ***P*=0.002**	25 (30.9)	56 (69.1)	31 (38.3)	50 (61.7), ***P*=0.001**	42 (51.9)	36 (48.1), ***P*=0.038**
III-IV	52	9 (17.3)	43 (82.7)	20 (38.5)	32 (61.5)	6 (11.5)	46 (88.5)	18 (34.6)	34 (65.4)
**Smoker**									
No	65	21 (32.3)	44 (67.7)	23 (35.4)	42 (64.6)	15 (18.5)	29 (81.5)	29 (44.6)	36 (55.4)
Yes	44	14 (31.8)	30 (68.2)	15 (34.1)	29 (65.9)	14 (34.1)	21 (65.9)	20 (45.5)	24 (54.5)
**Type^b^**									
SCC	17	7 (41.2)	10 (58.8)	2 (11.8)	15 (88.2), ***P*=0.045**	6 (35.3)	11 (64.7)	9 (52.9)	8 (47.1)
ADC	113	34 (30.1)	79 (69.9)	41 (36.3)	72 (63.7)	30 (26.5)	83 (73.5)	50 (44.2)	63 (55.8)
**Type^c^**									
I-II	84	28 (33.3)	56 (66.7)	28 (33.3)	56 (66.7)	24 (28.6)	60 (71.4)	43 (51.2)	41 (48.8)*,* ***P*=0.048**
III-IV	49	15 (30.6)	34 (69.4)	17 (34.7)	32 (65.3)	13 (26.5)	36 (73.5)	17 (34.7)	36 (65.3)
**N stage^d^**									
0	66	29 (43.9)	37 (56.1), ***P*=0.004**	19 (28.8)	47 (71.2)	26 (39.4)	40 (60.6), ***P*=0.003**	34 (51.5)	32 (48.5)
1-2	67	14 (20.9)	53 (79.1)	26 (38.8)	41 (61.2)	11 (16.4)	56 (83.6)	26 (38.8)	41 (61.2)
**M stage^e^**									
0	121	43 (35.5)	78 (64.5), ***P*=0.011**	40 (33.1)	81 (66.9)	36 (29.8)	85 (70.2)	56 (46.3)	65 (53.7)
1	11	0 (0.00)	11 (100.0)	4 (36.4)	7 (63.6)	1 (9.10)	10 (90.9)	4 (36.4)	7 (63.6)
**Shh**									
Low	37	21 (56.8)	16 (43.2), ***P*<0.001**						
High	96	22 (22.9)	74 (77.1)						
**CD31**									
Low	60	30 (50.0)	30 (50.0), ***P*<0.001**	18 (30.0)	42 (70.0)	24 (40.0)	36 (60.0), ***P*=0.004**		
High	73	13 (17.8)	60 (82.9)	27 (36.5)	47 (63.5)	13 (17.8)	60 (82.2)		
**YY1**									
Low	45	16 (35.6)	29 (64.4)						
High	88	31 (35.2)	57 (64.8)						

^aa^The protein expression pattern was defined as low cancer baseline expression (low) or high expression (high). The data were analyzed by Pearson χ^2^ test. *P* values with significance are shown as superscripts (*P* < 0.05). ^b^ADC, adenocarcinoma; SCC, squamous cell carcinoma; ^c^T Stage: tumor size; ^d^ N Stage: lymph node metastasis; ^e^M Stage: distant metastasis.

**Table 2:** Cox regression analysis of risk factors for cancer-related death in 133 lung cancer patients

Characteristics	Univariate analysis		Multivariate analysis	
	HR^a^ (95% CI^b^)	*P*-value^c^	HR^a^ (95% CI^b^)	*P*-value^c^
**ZNF322A expression**				
Low expression	1.00		- *i*	
High expression	2.781 (1.449-5.339)	**0.002**	- *i*	- *i*
**YY1 expression**				
Low expression	1.00		- *i*	
High expression	0.914 (0.536-1.559)	0.741	- *i*	- *i*
**Shh expression**				
Low expression	1.00		- *i*	
High expression	1.227 (0.686-2.195)	0.490	- *i*	- *i*
**CD31**				
Low expression	1.00		- *i*	
High expression	2.021 (1.185-3.445)	**0.010**	- *i*	- *i*
**ZNF322A/CD31^d^**				
Low expression (-/-)	1.00		1.00	
Others (-/+; +/-)	4.915 (1.699-14.22)	**0.003**	3.962 (1.352-11.60)	**0.012**
High expression (+/+)	5.869 (2.084-16.52)	**0.001**	3.952 (1.358-11.50)	**0.012**
**Age expression**				
<65 year-old	1.00		- *i*	
>65 year-old	0.797 (0.481-1.319)	0.377	- *i*	- *i*
**Gender**				
Female	1.00		- *i*	
Male	1.421 (0.852-2.370)	0.178	- *i*	- *i*
**Smoking habit**				
Non-smoker	1.00		- *i*	
Smoker	1.665 (0.948-2.922)	0.076	- *i*	- *i*
**Type^e^**				
SCC	1.00		- *i*	
ADC	0.636 (0.323-1.254)	0.191	- *i*	- *i*
**Stage**				
Stage I-II	1.00		1.00	
Stage III-IV	2.753 (1.656-4.577)	**<0.001**	1.364 (0.620-2.998)	0.440
**T stage^f^**				
Stage 1-2	1.00		- *i*	
Stage 3-4	1.345 (0.803-2.254)	0.260	- *i*	- *i*
**N stage^g^**				
N0	1.00		1.00	
≥N1	2.681 (1.559-4.610)	<0.001	1.532 (0.688-3.410)	0.296
**M stage^h^**				
M0	1.00		1.00	
≥M1	3.503 (1.752-7.003)	<0.001	1.969 (0.933-4.156)	0.075

^a^HR, Hazard ratio. ^b^CI, Confidence interval. ^c^Bold values indicate statistical significance (*P* < 0.05). ^d^ZNF322A expression is shown before the slash followed by CD31 expression. -, low expression; +, high expression. ^e^ADC, Adenocarcinoma; SCC, Squamous cell carcinoma. ^f^T Stage: tumor size. ^g^N Stage: lymph node metastasis. ^h^M Stage: distant metastasis. ^i^The variables without significant HR in the univariate analysis were not included in the multivariate analysis.

## Abbreviations

ADC: adenocarcinoma
ChIP: chromatin-immunoprecipitation sequencing
CM: conditioned medium
HUVEC: human umbilical vein endothelial cell
OS: overall survival
PFS: progression-free survival
SCC: squamous cell carcinoma
Shh: sonic hedgehog
TF: transcription factor
YY1: Yin Yang 1
