# Supplementary material for: Dysregulated Kras/YY1/ZNF322A/Shh transcriptional axis enhances neo-angiogenesis to promote lung cancer progression
Source: Theranostics. 2020 Aug 8;10(22):10001–15. doi: 10.7150/thno.47491 (PMC7481419; doi:10.7150/thno.47491)
Supplement: Supplementary file 1 — Supplementary figures and tables. [file thnov10p10001s1.pdf]

Supplemental figures and legends

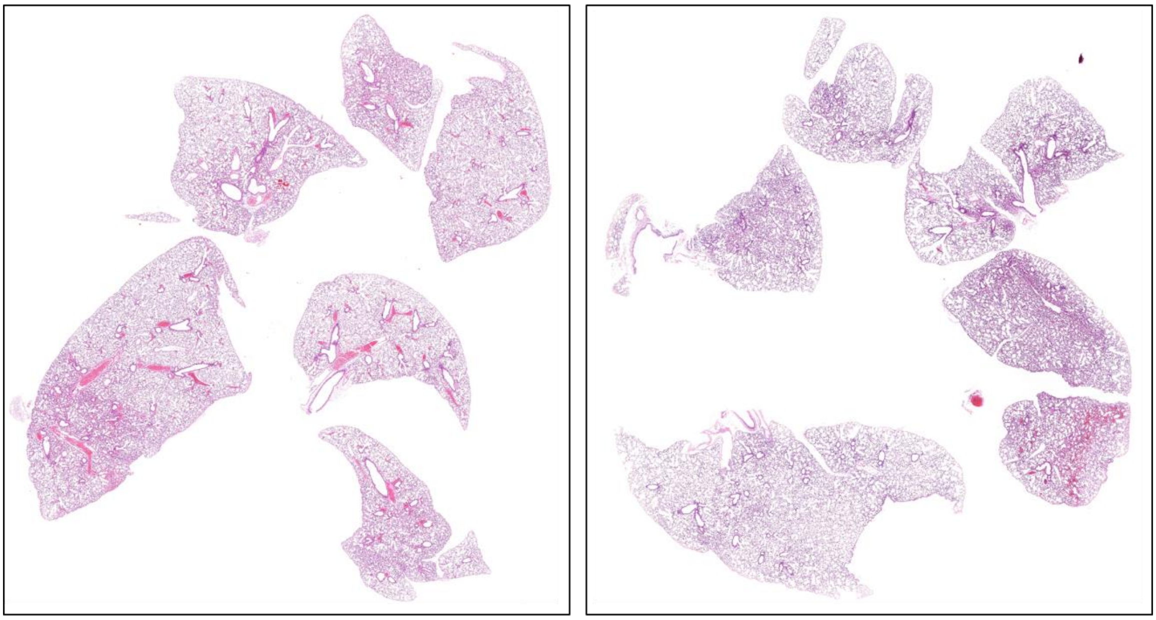

znf322a transgenic mouse 1 | *znf322a* transgenic mouse 2

Figure S1. *znf322a* transgenic mice did not show spontaneous lung tumorigenesis *in vivo.* The mice were sacrificed and lung lobes were removed for histopathology. H&E staining of histopathological section were viewed and scanned images of the entire lung were collected. Two representative mice images are shown. A total of 30 mice were followed for two years to ascertain the tumor formation status.

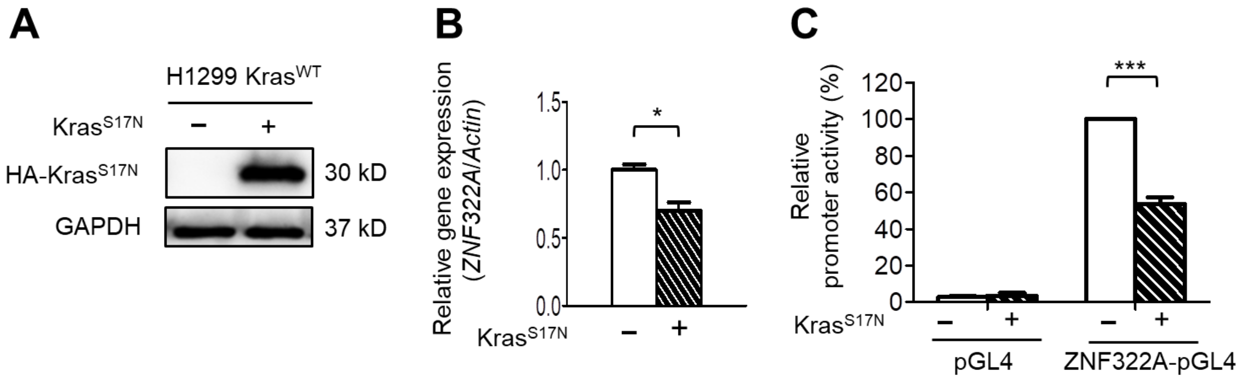

Figure S2. Dominant-negative Kras^{S17N} mutation attenuated *ZNF322A* transcription. **A,** Immunoblotting analysis confirmed that dominant-negative Kras (HA-Kras^{S17N}) was overexpressed in H1299 cells. **B,** *ZNF322A* mRNA expression was reduced upon Kras^{S17N} overexpression in H1299 cells. **C,** *ZNF322A*-pGL4 promoter activity was reduced upon Kras^{S17N} overexpression in H1299 cells. Data were mean ± SEM and normalized to the control group (-). *, P<0.05; ***, P<0.001.

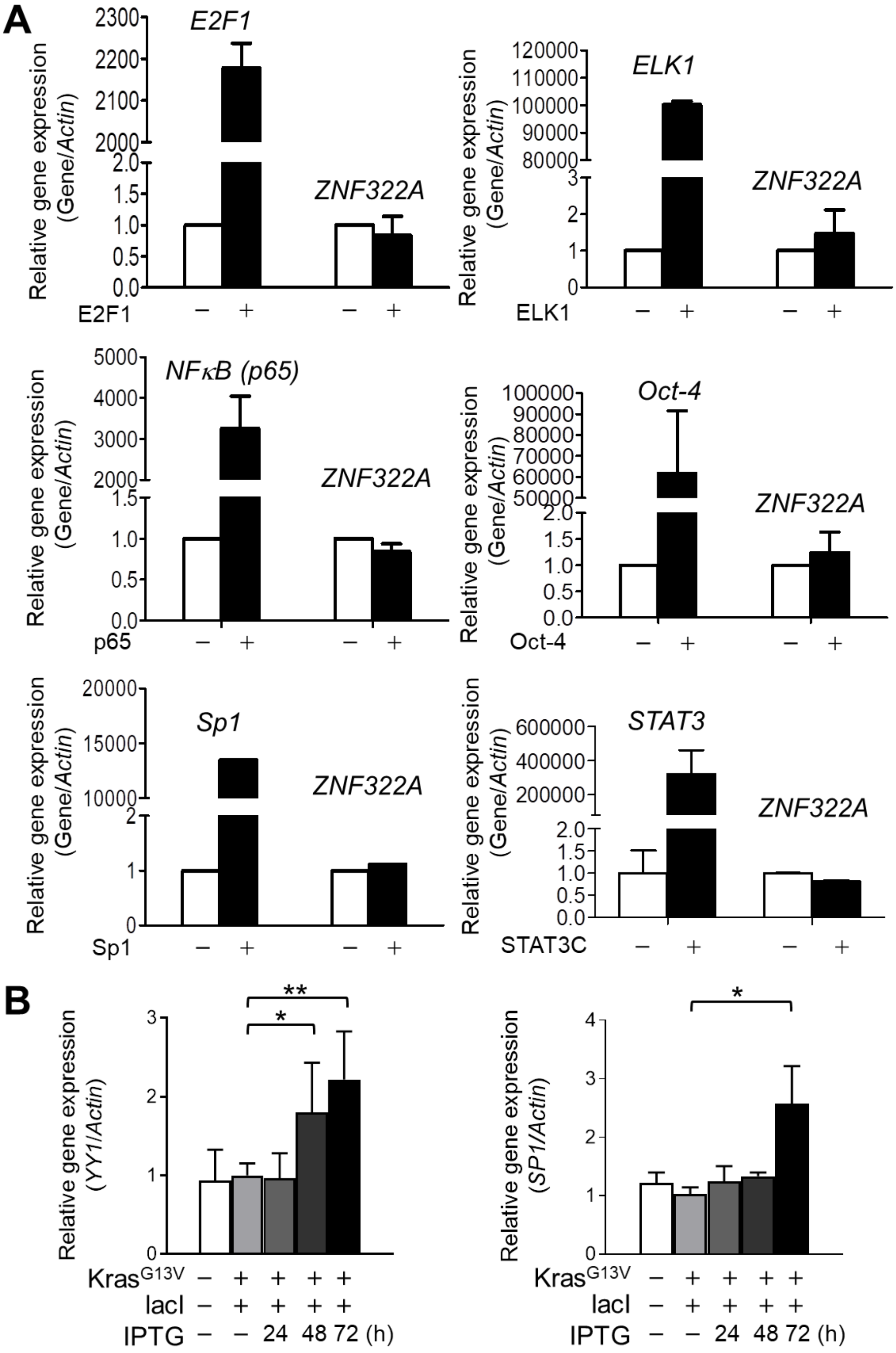

Figure S3. The tested transcription factors in H460 cells. **A,** RT-qPCR analysis showed that overexpression of E2F1, ELK1, NFκB (p65), Oct4, Sp1 or STAT3 did

not affect *ZNF322A* mRNA expression in H460 cell. **B,** RT-qPCR revealed that *YY1* mRNA expression was upregulated by $Kras^{G13V}$ in a dose-dependent manner (*left*), while *Sp1* mRNA expression was induced by $Kras^{G13V}$ overexpression at the 72 h time point in H460 cells (*right*). Target gene expression levels were normalized to the expression level of housekeeping gene *β-actin.* Data were then normalized to the empty vector control (-) group. Data were mean ± SEM and normalized to the control group. *, $P<0.05$; **, $P<0.01$.

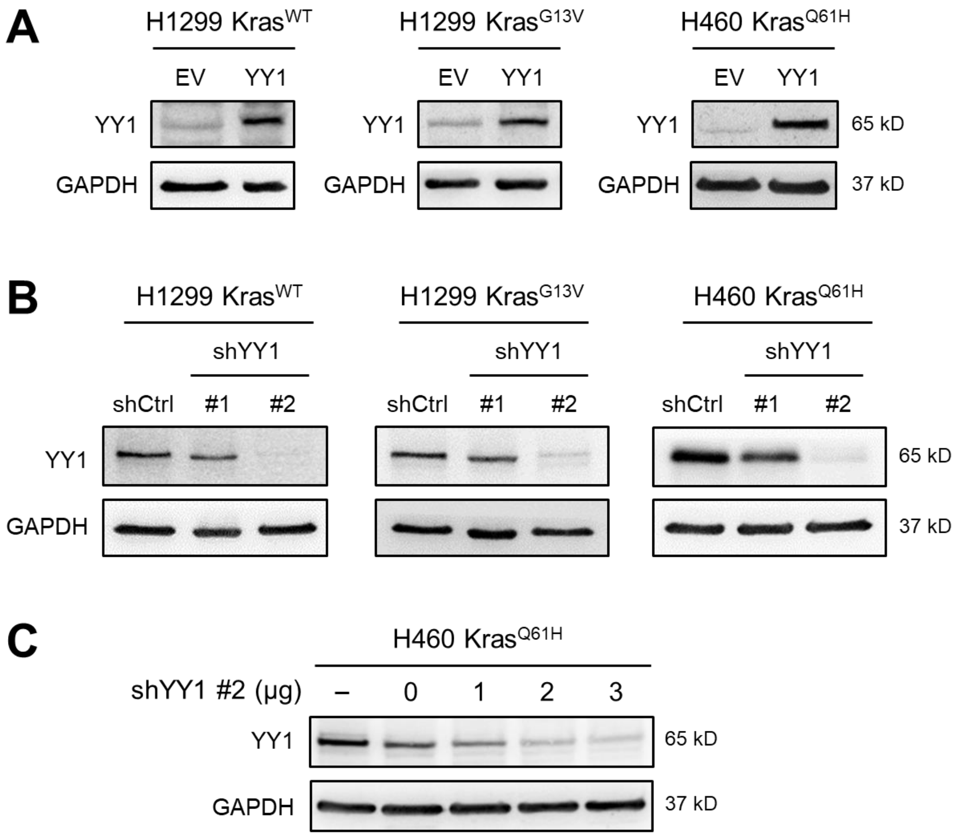

Figure S4. The protein levels of the YY1 overexpression and knockdown in tested cell lines. **A,** Western blotting confirmed that YY1 was successfully overexpressed in H1299 $Kras^{WT}$, H1299 $Kras^{G13V}$ and H460 $Kras^{Q61H}$ cells. **B** and **C,** Western blotting revealed that the #2 shYY1 clone showed a better knockdown efficiency than the #1 clone (B). The #2 clone exerted a dose-dependent knockdown of YY1 in H460 $Kras^{Q61H}$ cells (C). Therefore, the #2 clone was used for all YY1 knockdown experiments.

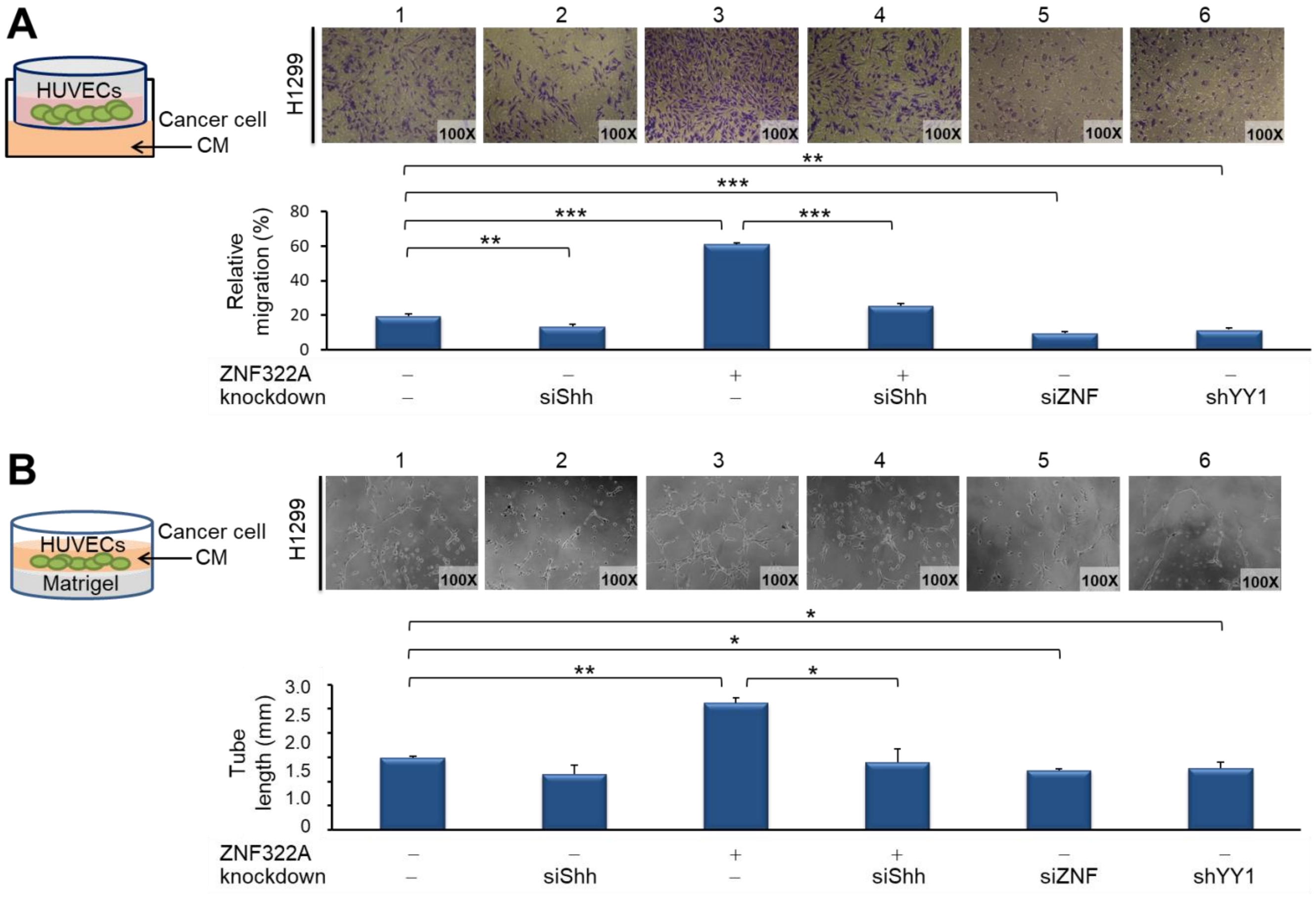

Figure S5. YY1/ZNF322A/Shh axis regulated transwell migration assay and tumor formation abilities of HUVECs. **A** and **B**, Transwell migration assay (A) and tumor formation assay (B) showed that CM derived from si*Shh* in ZNF322A overexpressing (*group 4*) cells inhibited HUVECs migration ability or tumor formation ability compared with CM from ZNF322A-overexpressed (*group 3*) H1299 lung cancer cells. Knockdown of ZNF322A (*group 5*) or YY1 (*group 6*) in H1299 lung cancer cells also attenuated HUVECs migration and tumor formation abilities (panels 5 and 6). siShh was included for comparison (*group 2*). The migration ability was monitored at 24 h with each group quantified by comparising with initial seeding number of HUVECs (*Upper left*). The tube formation was monitored at 6-8 h with each group quantified for the tube length (*Lower left*). Data are presented as mean ± SEM. *P*-values determined using two-tailed Student’s *t*-test. *$P < 0.05$; ** $P < 0.01$; *** $P < 0.001$.

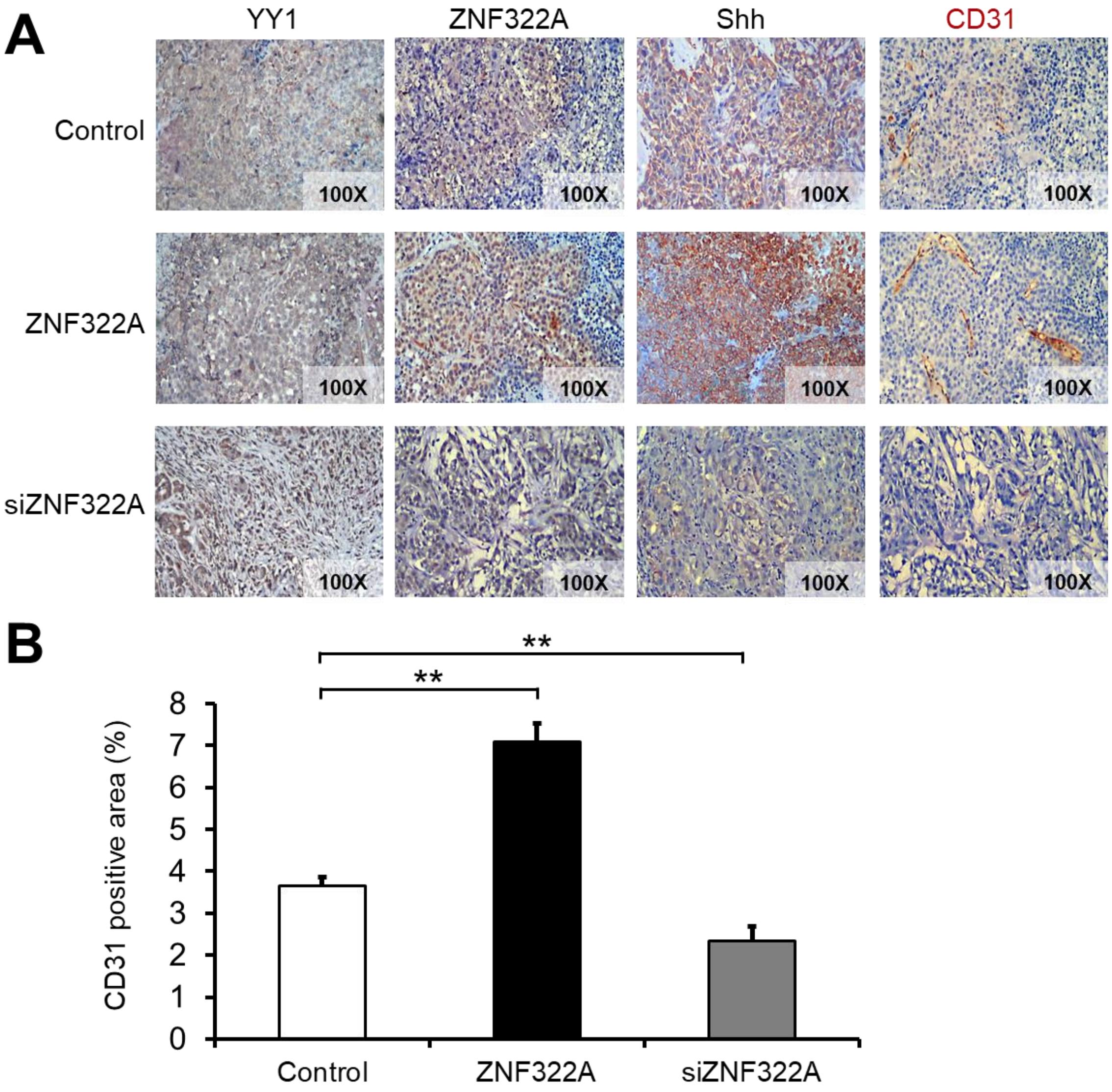

Figure S6. CD31 angiogenesis marker and staining of YY1, ZNF322A and Shh by IHC in tumor xenograft of H460 $Kras^{Q61H}$ lung cancer cells. **A,** The level and distribution of CD31, an endothelial cells marker, and Shh increased in tumor xenograft of ZNF322A-overexpressing H460 lung cancer cells while decreased in siZNF322A xenograft compared to the control H460 $Kras^{Q61H}$ group. **B,** The angiogenesis of each group was measured by percentage of the area of CD31-positive stained cells in the detected regions. *P* values were calculated by two-tailed *t*-test. Data were mean ± SEM. **, *P*<0.01.

Supplemental tables

Table S1. The plasmids and their characteristics used in the study.

Plasmid	Target	Insert (bp)	Function	Source
HA-pOPI3-Kras^{G13V}	Kras^{G13V}	685	Overexpression	From Dr. H-S Liu
pHβlacI	lacI	1100	Overexpression	From Dr. H-S Liu
HA-pcDNA3.1-Kras^{S17N}	Kras^{S17N}	670	Overexpression	From Dr. H-S Liu
pCMV-SPORT6-E2F1	E2F1	1314	Overexpression	GenDiscovery
pLAS2w-Elk1	Elk1	1286	Overexpression	From Dr. J-Y Liu
HA-pCDNA3-Sp1	Sp1	2358	Overexpression	From Dr. J-J Hong
pCMV4-NFκB (p65)	NFκB (p65)	2500	Overexpression	From Dr. Y-N Teng
pPyCAGIP-Flag-Oct4	Oct4	1083	Overexpression	From Dr. Ying Jin
pCMV-HA	None	–[a]	Vector control	Clontech
HA-ZNF322A	Wild type ZNF322A	1083	Overexpression	Homemade[b]
pLHCX-YY1	YY1	1242	Overexpression	From Dr. H-L Hsu
pBS-Shh	Sonic hedgehog	749	Overexpression	Addgene
pGL4-vector	None	–[a]	Vector control	Promega
ZNF322A-pGL4	*ZNF322A* promoter	752	Promoter activity assay	Homemade[b]
Del-ZNF322A-pGL4	*ZNF322A* promoter with deletion	352	Promoter activity assay	Homemade[b]
Shh-pGL4	*Shh* promoter	977	Promoter activity assay	Homemade[b]
Mut-Shh-pGL4	*Shh* promoter with mutation	977	Promoter activity assay	Homemade[b]
Renilla	None	–[a]	Vector control	Promega

[a] The plasmid is used as a backbone vector therefore has no inserted fragment.

[b] Human ZNF322A cDNA was PCR-amplified and cloned into pCMV-HA to generate HA-tagged ZNF322A expression construct. For ZNF322A-pGL4 plasmid, human *ZNF322A* promoter region (-529 to +223) was PCR-amplified and cloned into pGL4 expression vector to generate *ZNF322A* promoter luciferase reporter plasmid. For Del-ZNF322A-pGL4 plasmid, human *ZNF322A* promoter region (-129 to +223) was PCR-amplified and cloned into pGL4 expression vector to generate YY1 element-deleted *ZNF322A* promoter luciferase reporter plasmid. For Shh-pGL4 plasmid, human *Shh* promoter region (-678 to +298) was PCR-amplified and cloned into pGL4 expression vector to generate *Shh* promoter luciferase reporter plasmid. For Mut-Shh-pGL4 plasmid, human *Shh* promoter region (-678 to +298) was PCR-amplified, among +87 to +89 (GTT→AGG) was mutated and cloned into pGL4 expression vector to generate mutant *Shh* luciferase reporter plasmid.

Table S2. The primers used in the current study.

**Gene**	**Primer**	Sequences (5’→ 3’)	**Application**[a]	**PCR size (bp)**	**Tm (°C)**
Actin mRNA	Forward	GGC GGC ACC ACC ATG TAC CCT	RT-qPCR	202	60
	Reverse	AGG GGC CGG ACT CGT CAT ACT			
ZNF322A mRNA	Forward	GTG GTC TGC GTG TGA GAG TGG C	RT-qPCR	226	60
	Reverse	TTC TGA CGC ATG GGG AGG GCT			
YY1 mRNA	Forward	TTG CTA GAA TGA AGC CAA GAA AAA	RT-qPCR	101	60
	Reverse	GGC CGA GTT ATC CCT GAA CAT			
Shh mRNA	Forward	CCC AAT TAC AAC CCC GAC ATC	RT-qPCR	142	60
	Reverse	TCA CCC GCA GTT TCA CTC CT			
E2F1 mRNA	Forward	GCA GAG CAG ATG GTT ATG GTG AT	RT-qPCR	125	60
	Reverse	GGG CAC AGG AAA ACA TCG AT			
ELK1 mRNA	Forward	TCACGGGATGGTGGTGAATT	RT-qPCR	146	60
	Reverse	ACCTTGCGGATGATGTTCTTG			
Sp1 mRNA	Forward	GCG AGA GGC CAT TTA TGT GT	RT-qPCR	189	58
	Reverse	GGC CTC CCT TCT TAT TCT GG			
NFκB (p65) mRNA	Forward	GGC CTT GCT TGG CAA CAG	RT-qPCR	100	60
	Reverse	CAC AGG TAT GCC CTG GTT CA			
Oct4 mRNA	Forward	CGA AAG AGA AAG CGA ACC AG	RT-qPCR	157	60
	Reverse	GCC GGT TAC AGA ACC ACA CT			
c-Jun mRNA	Forward	TTT CAG GAG GCT GGA GGA AGG GG	RT-qPCR	107	60
	Reverse	AAT GGT CAC AGC ACA TGC CAC T			
HIF-1α mRNA	Forward	CCA TTA CCC ACC GCT GAA A	RT-qPCR	195	60
	Reverse	GGG ACT ATT AGG CTC AGG TGA ACT T			
VEGFA mRNA	Forward	TAC CTC CAC CAT GCC AAG TG	RT-qPCR	100	60
	Reverse	TGC GCT GAT AGA CAT CCA TGA			
IL-8 mRNA	Forward	CCT TTC CAC CCC AAA TTT ATC A	RT-qPCR	160	60
	Reverse	CCC TCT TCA AAA ACT TCT CCA CAA			

**Gene**	**Primer**	Sequences (5'→ 3')	**Application[a]**	**PCR size (bp)**	**Tm (°C)**
NOTCH1 mRNA	Forward	GGC ACG ACG CCA CTG ATC	RT-qPCR	199	60
	Reverse	CCC TGT TGT TCT GCA TAT CTT TGT			
TGFβ2 mRNA	Forward	AAA GCC AGA GTG CCT GAA CAA	RT-qPCR	150	60
	Reverse	AAC AGC ATC AGT TAC ATC GAA GGA			
COL15A1 mRNA	Forward	GGA AAA AAT TAC AGC TGG GAG AAC	RT-qPCR	155	60
	Reverse	GTT CAG AGC AGC CAA ATG CA			
ZNF322A-promoter	Forward	TGA TTT ACT CTT AGC TGA TT	Construction	752	60
	Reverse	AGA CTC TGC GAT AGT AGA TC			
Del-ZNF322A-promoter	Forward	AAA ACG CCG AGG GCT CCC AG	deletion	352	70
	Reverse	AGA CTC TGC GAT AGT AGA TC			
ZNF322A-promoter-ChIP	Forward	CTT TCT CTC GCT GCT GTT TTC TC	ChIP-qPCR	100	60
	Reverse	GGC AGC GCT GTC ATC CA			
Shh-promoter	Forward	GCG CGG TAC CCG GGG GTT TAA AC	Construction	977	62
	Reverse	GCG CCT CGA GTG AGT CAT CAG CC			
Mut-Shh-promoter	Forward	GGG CAG GGG AGG CGG GAA G	Site-direct mutagenesis	–[b]	62
	Reverse	CTT CCC GCC TCC CCT GCC C			
Shh-promoter-ChIP	Forward	GCG CAA AGC GCA AGA GAG AGC G	ChIP-qPCR	210	62
	Reverse	GCG CTT TTG GGG TGC CTC CTC T			

[a] qRT-PCR, quantitative reverse-transcriptase polymer chain reaction; ChIP-qPCR, quantitative chromatin-immunoprecipitation coupled with polymer chain reaction.

[b] Not applicable.

Table S3. Antibodies and their reaction conditions in the study.

Target	KD	Raised In	Application [a]	Dilution	Source	Catalog No.
ZNF322A	44	Mouse	IHC<br>Western blotting	1:100<br>1:1000	Homemade [b]	– [c]
HA-tag	– [c]	Mouse	Western blotting<br>ChIP	1:1000<br>1:50	Bioman	HAT001M
GAPDH	37	Mouse	Western blotting	1:1000	Santa Cruz	sc-32233
YY1	65	Rabbit	IHC<br>Western blotting<br>ChIP	1:200<br>1:200<br>1:100	Abcam	ab38422
Shh	55	Rabbit	IHC<br>Western blot	1:500<br>1:1000	Abcam	Ab53281
CD31	100	Rabbit	IHC	1:300	Abcam	ab28364

[a] IHC: immunohistochemistry; ChIP: chromatin immunoprecipitation.

[b] Homemade, Anti-ZNF322A was generated by Kelowna International Scientific Inc, Taiwan using synthetic peptides (CNVSEKGLELSPPHASE).

[c] –, Information is not applicable.
